# Differential epithelial and stromal protein profiles in cone and non-cone regions of keratoconus corneas

**DOI:** 10.1038/s41598-019-39182-6

**Published:** 2019-02-27

**Authors:** Gary Hin-Fai Yam, Matthias Fuest, Lei Zhou, Yu-Chi Liu, Lu Deng, Anita Sook-Yee Chan, Hon Shing Ong, Wei-Boon Khor, Marcus Ang, Jodhbir S. Mehta

**Affiliations:** 1 Tissue Engineering and Stem Cell Group, Singapore Eye Research Institute, Singapore, Singapore; 2 Ophthalmology and Visual Science Academic Clinical Research Program, Duke-National University of Singapore Graduate Medical School, Singapore, Singapore; 3 Department of Ophthalmology, RWTH Aachen University, Aachen, Germany; 4 Ocular Proteomics Platform, Singapore Eye Research Institute, Singapore, Singapore; 5 Department of Ophthalmology, Yong Loo Lin School of Medicine, National University of Singapore, Singapore, Singapore; 6 Department of Statistics and Applied Probability, National University of Singapore, Singapore, Singapore; 7 Ophthalmic Pathology Platform, Singapore Eye Research Institute, Singapore, Singapore; 8 Singapore National Eye Centre, Singapore, Singapore; 9 School of Material Science and Engineering, Nanyang Technological University, Singapore, Singapore

## Abstract

Keratoconus (KC) is an ectatic corneal disease characterized by progressive thinning and irregular astigmatism, and a leading indication for corneal transplantation. KC-associated changes have been demonstrated for the entire cornea, but the pathological thinning and mechanical weakening is usually localized. We performed quantitative proteomics using Sequential Windowed Acquisition of All Theoretical Fragment Ion Mass Spectrometry (SWATH-MS) to analyze epithelial and stromal changes between the topographically-abnormal cone and topographically-normal non-cone regions of advanced KC corneas, compared to age-matched normal corneas. Expression of 20 epithelial and 14 stromal proteins was significantly altered (≥2 or ≤0.5-fold) between cone and non-cone in all 4 KC samples. Ingenuity pathway analysis illustrated developmental and metabolic disorders for the altered epithelial proteome with mitochondrion as the significant gene ontology (GO) term. The differential stromal proteome was related to cellular assembly, tissue organization and connective tissue disorders with endoplasmic reticulum protein folding as the significant GO term. Validation of selected protein expression was performed on archived KC, non-KC and normal corneal specimens by immunohistochemistry. This is the first time to show that KC-associated proteome changes were not limited to the topographically-thinner and mechanically-weakened cone but also non-cone region with normal topography, indicating a peripheral involvement in KC development.

## Introduction

Keratoconus (KC) is an asymmetric corneal ectatic disorder characterized by progressive focal thinning, that leads to myopia and irregular astigmatism with impaired visual acuity^1,2^. Corneal steepening and protrusion, with an eccentric thin conical apex is the typical clinical presentation, whereas central scarring is seen in many advanced cases^3^. The incidence of KC is estimated to range from 1:400 to 1:2,000 people worldwide^4^. Ethnic differences of KC incidence have also been reported^5–7^. It affects both genders at puberty to early mid-life, hence it significantly impacts young, working individuals and poses a considerable socioeconomic burden to the society^3^. About 20% of KC patients require corneal transplantation and it is one of the leading indications for corneal grafting in the US and worldwide^8,9^.

KC is classically defined as a non-inflammatory corneal degeneration due to the lack of cellular infiltration and neovascularization^10^. However, increasing evidence has revised this theory and the etiology of KC is still not completely understood^1^. The etiology of KC is multifactorial, including genetic, biomechanical and environmental^11^. The detection of a positive family history in approximately 10% of KC cases and a high correlation among monozygotic twins have suggested a genetic etiology^12^. Linkage and genome-wide association studies have identified possible loci and gene variants, yet they remain to be validated in larger cohorts^1,13–16^. External factors, such as contact lens wear, eye rubbing and ultraviolet (UV) light  exposure, potentially induce corneal microtrauma and trigger the production of inflammatory mediators^17^. Subjects with a history of ocular allergy and atopy are also at high risk of developing KC^18,19^. In addition, epigenetic factors could influence the complex etiology of KC, in particular the association between genome and the environment^20^. MicroRNA screening on impression cytology samples has shown several down-regulated microRNAs in KC epithelia^21^. Mutations of miR-184 have been reported to be associated with familial severe KC^22,23^.

KC pathophysiology is largely defined into: (i) altered stromal composition, e.g. altered collagen and proteoglycan content causing a reduced stromal mass, lamellar slippage and reduced interweaving of collagen^24,25^; (ii) enzymatic imbalance causing stromal degradation^26^; (iii) expression of inflammation-related mediators to regulate protease cascades (e.g. tissue plasminogen activator and metalloproteinases, MMPs) leading to stromal matrix changes^27–31^ and (iv) oxidative stress-induced protein denaturation causing damage to DNA and mitochondrial functions, inducing apoptosis and stromal degradation^32–34^.

Tear film studies using proteomics and bioinformatics have shown altered proteins belonging to families of proteinases, inflammatory cytokines, cell adhesion molecules, glycoproteins and transporters in samples from KC patients, compared to controls^29,30,35–39^. The findings of elevated interleukin-6, tumor necrosis factor-α and matrix metalloproteinase-9 (MMP-9) in KC tear samples have highlighted the cytokine imbalance and inflammatory mediators on the ocular surface^28,40,41^. Studies on aqueous humor protein profiles in KC patients have implicated altered biological processes, including the regulation of proteolysis and response to hypoxia and oxidative stress^42^. A recent report on saliva proteomics has identified significant hormonal metabolite changes (including IL16, myoinositol and 1-methyl-histidine) associated with pro-inflammatory processes in KC versus healthy control subjects^43^. However, changes in tear film, aqueous humor and saliva content may not necessarily reflect the intracorneal processes, in particular the structural thinning. Recent studies on isolated KC epithelia and stroma have illustrated altered expression of cytokeratins and cytoskeleton, matrix components and regulatory proteins, suggesting that various degenerative pathways in association with inflammation, changes of innate immune functions, oxidative stress, abnormal mitochondrial functions, and cell death occur in KC^25,44–47^.

KC severity is related to the degree of topographic deformation (thinning and protrusion) in a localized cone region, of which the apex is often inferiorly decentered from the pupil axis^48^. Outside this ectatic zone, the non-cone region is topographically normal^49^. Whether the pathological cellular changes of the corneal epithelium and stroma are only limited to the areas of topographical abnormality and central thinning, or are widespread to the entire cornea is unknown.

**Figure 1:**
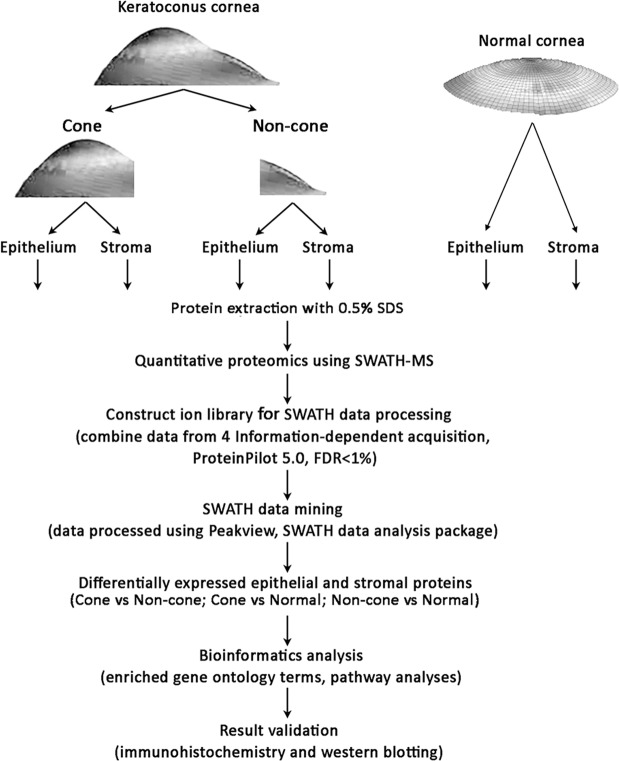
Quantitative proteomic workflow of keratoconus cornea (cone and non-cone regions) with separated corneal epithelium and stroma versus normal corneal samples using a SWATH-MS approach.

We conducted a comprehensive screening of protein expression in the corneal epithelia and stroma of the respective cone and non-cone regions from four KC patients using a robust high-performance, high-sensitivity, label-free liquid chromatography with tandem mass spectrometry (LC-MS/MS) linked to SWATH (Sequential Windowed Acquisition of All Theoretical Fragment Ion Mass Spectra) (SWATH-MS)^50,51^. In this pilot study, with paired cone versus non-cone comparison and with age-matched normal controls, we identified candidate epithelial and stromal proteins that were differentially expressed in tissues. We examined the enriched gene ontology (GO) terms and putative signalling pathway changes using web-based DAVID (Database for Annotation, Visualization and Integrated Discovery) Bioinformatics Resources and Ingenuity Pathway Analysis (IPA, Qiagen). Figure 1 shows the schematic summary of this study. Selected proteins were validated on additional archived KC samples, and compared to corneal scar samples (non-KC cases) and normal corneal tissues, by immunohistochemistry and western blotting.

## Results

### Clinical analysis of KC patients

**Figure 2:**
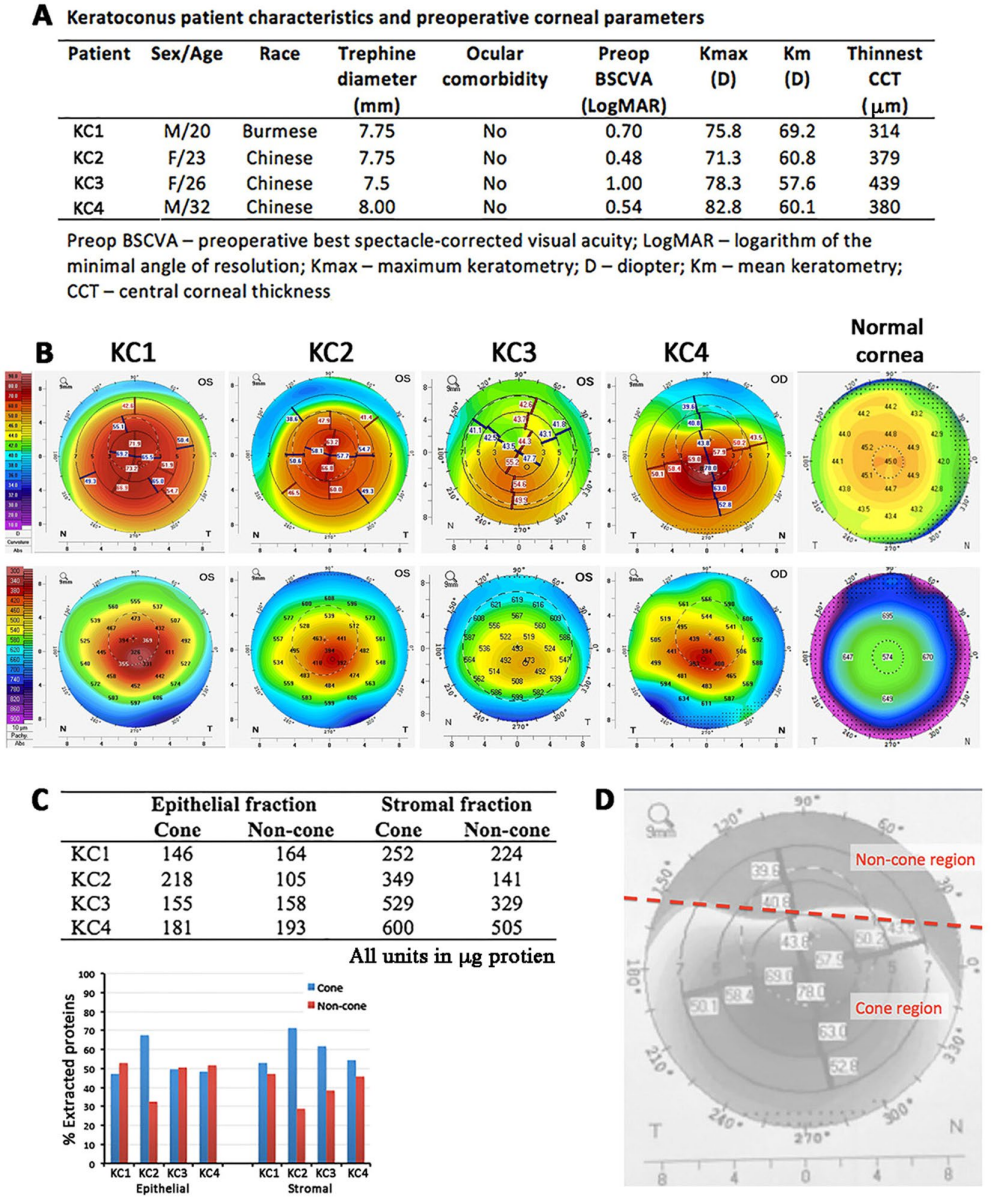
Patient information and cornea samples. (**A**) Patient demographic information and preoperative corneal parameters. (**B**) Pentacam images of keratoconus corneas displaying anterior curvature (measured in diopters D, top line) and corneal thickness maps (measured in µm, bottom line) to indicate the position of cone areas, compared to normal corneal topography. The numbers from 0 to 8 on the x and y axis as well as the 3, 5 and 7 rings in the top row indicate the diameter in mm from the corneal center/apex. (**C**) Extracted protein quantities of epithelial and stromal fractions from KC cone and non-cone samples (patient 1 to 4).(**D**) A schematic diagram showing the separation of cone and non-cone regions according to the pre-operative topographic data.

The clinical characteristics of 4 patients with advanced KC are summarized in Fig. 2A. The mean pre-operative best spectacle-corrected visual acuity (BSCVA) was 0.68 ± 0.23 (range 1.0 to 0.48) LogMAR, the mean maximum curvature power (Kmax) was 77.1 ± 4.8 (patient range 71.3 to 82.8 diopters, D, versus normal 42.0 to 46.0 D), the mean overall keratometric values (Km) was 61.9 ± 5.0 D (patient range 57.6 to 69.2 D versus normal 42.0 to 45.0 D) and the mean thinnest central corneal thickness (CCT) was 378 ± 51 μm (patient range 314 to 439 μm versus normal 524 ± 25 μm). The pre-operative topographic evaluation of KC corneas by Pentacam-Scheimpflug imaging (Pentacam, Oculus Inc., Lynnwood, WA, USA) displayed corneal thickness and anterior curvature maps to indicate the position of the thinnest point (cone apex) compared to normal cornea (Fig. 2B).

### KC cone and non-cone epithelial and stromal protein samples

SDS-extracted protein fractions of cone epithelium, cone stroma, non-cone epithelium and non-cone stroma from 4 KC corneas and normal epithelium and normal stroma from 2 age-matched control corneas were quantified. Without pooling samples, this preserved the unique protein features of individual KC cornea. Except for KC2 samples, the other KC pairs (KC1, 3, and 4) had similar extracted protein levels from cone and non-cone tissues (48.3 ± 1.2% for cone epithelial and 56.3 ± 4.7% for cone stromal) (Fig. 2C). KC2 had ~2-fold more epithelial and stromal proteins extracted from cone than in non-cone region (Supplemental Fig. S1).

### Proteins identified and quantified in KC corneal tissues by SWATH-MS

The experimental scheme is depicted in Fig. 1. A constant of 100 μg protein per sample were loaded to SWATH-MS analysis. A spectral library contained 2980 distinct proteins (1997 epithelial and 983 stromal) identified with >95% confidence and global false discovery rate (FDR) <1%. Among them, 1626 epithelial and 799 stromal proteins were quantifiable (Supplemental Data File S1). There were 587 proteins expressed in both epithelial and stromal samples (Supplemental Fig. S2). The strong concordance of technical replicates was verified by using the standard yeast digest sample (Promega, MA, USA) (R^2^ = 0.998, from 2 individual runs, Supplemental Fig. S3).

### Epithelial proteomes

#### KC cone versus normal corneal epithelial tissue

**Figure 3:**
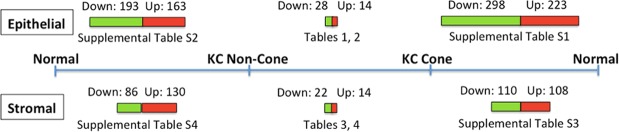
A bar chart summarizing the epithelial and stromal protein changes of KC non-cone versus normal, KC cone versus normal and KC cone versus non-cone samples. The bar lengths are in proportion to number of proteins with significant changes (*p* < 0.05); red-colored bars represent up-regulation and green-colored bars represent down-regulation. The tables showing the pathway results for each comparison are stated. Note: both normal are the same samples.

**Figure 4:**
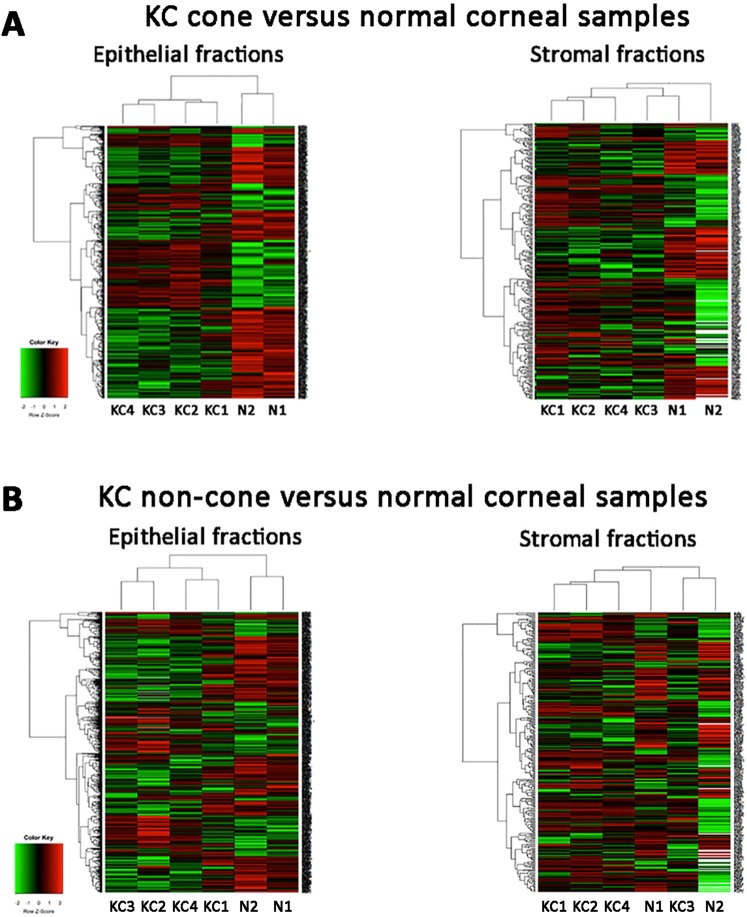
Heat maps of an unsupervised hierarchical clustering of proteins with fold changes (>2 and <0.5). The colors in the map displayed the relative expression values. Green indicated the lowest expression, black for the intermediate expression, and red for the highest expression. The numerical values gave the actual values on a log2 scale, which were associated with each color. The color scale bar is shown at the lower left corner. (**A**) KC cone epithelium and stroma were compared to normal corneal samples. (**B**) KC non-cone epithelium and stroma were compared to normal corneal samples.

A list of 223 proteins were significantly upregulated (*p* < 0.05) and 298 significantly downregulated in all 4 KC cone samples compared to normal corneal epithelia (Fig. 3; Supplemental Data File S1 and data of mean fold changes in Supplemental Data File S2A). Similar to other studies comparing entire KC and normal corneas, our data of cone versus normal showed increased epithelial proteins including vimentin, S100A4, KRT12 and 14, decorin, IL18 (interleukin-18); and reduced proteins included transketolase, pyruvate transferase, LOX (lysyl oxidase), ZO-1 and SOD (superoxide dismutase). Heat map analysis showed distinct clustering between KC cone and normal epithelial protein profiles (Fig. 4A). Using DAVID function annotation tools, the enriched GO terms predicted for these altered proteins were significantly associated to cell-cell adhesion (*p* < 0.001, Benjamini test; enrichment score EnS:20.1), mitochondrial electron transport (*p* = 0.013; EnS:4.3), nuclear-transcribed mRNA catabolic process, nonsense-mediated decay (*p* < 0.001; EnS:3.9), cell redox homeostasis (*p* < 0.001; EnS:3.3), mRNA splicing via spliceosome (*p* = 0.03; EnS:3.1), gluconeogenesis (*p* = 0.006; EnS:2.9), and negative regulation of endopeptidase activity (*p* = 0.03; EnS:2.2) (Supplemental Table S1). The significant KEGG pathways were oxidative phosphorylation (*p* = 0.002; EnS:5.3), Parkinson’s disease (*p* = 0.002; EnS:5.3), glycoxylate and dicarboxylate metabolism (*p* = 0.015; EnS:2.1) (Supplemental Table S1). Similar biological events were replicated using IPA method, including EIF2 signalling (*p* < 0.001), mitochondrial dysfunction (*p* < 0.001), oxidative phosphorylation (*p* < 0.001), caveolar-mediated endocytosis signalling (*p* < 0.001) and tight junction (*p* < 0.001). In the physiological and functional category, the affected pathways were (1) protein synthesis, gene expression, RNA post-transcriptional modification; (2) developmental disorders, hereditary disorders and metabolic diseases; (3) RNA molecular transport and trafficking; (4) dermatological disease and conditions and (5) cancer, organismal injury and abnormalities.

#### KC non-cone versus normal corneal epithelial tissue

A total of 163 proteins were significantly upregulated (*p* < 0.05) and 193 significantly downregulated in all 4 KC non-cone samples compared to normal corneal epithelia (Fig. 3; Supplemental Data File S2B). Heat map showed distinct dendrographic profiles between non-cone versus normal epithelium (Fig. 4B). Using DAVID bioinformatics, the significant enriched GO terms were identified to be cell-cell adhesion (*p* < 0.001, Benjamini test; enrichment score EnS:17.5), mitochondrial electron transport (*p* = 0.03; EnS:5.3), gluconeogenesis (*p* = 0.03; EnS:3.7), translation (*p* = 0.03; EnS:3.2), response to reactive oxygen species (*p* < 0.001; EnS:2.7) and actin cytoskeleton (*p* = 0.046; EnS:2.1) (Supplemental Table S2). The significant KEGG pathways were oxidative phosphorylation (*p* < 0.001; EnS:5.3), Parkinson’s disease (*p* < 0.001; EnS:5.3), glycolysis/gluconeogenesis (*p* < 0.001; EnS:3.7), cysteine and methionine metabolism (*p* = 0.03; EnS:3.1) and pyruvate metabolism (*p* = 0.03; EnS:3.1) (Supplemental Table S2). IPA replicated similar biological events, which were mitochondrial dysfunction (*p* < 0.001), phagosome maturation (*p* < 0.001), oxidative phosphorylation (*p* < 0.001), EIF2 signalling (*p* < 0.001) and glycolysis (*p* < 0.001). The affected physiological and functional categories included (1) cell morphology, cellular assembly, function and organization; (2) RNA post-transcriptional modification, cell death and survival (3) lipid metabolism; (4) cardiovascular disease, system development and (5) developmental, hereditary and metabolic disorders.

#### Differential expression of epithelial proteins in KC cone versus non-cone

**Figure 5:**
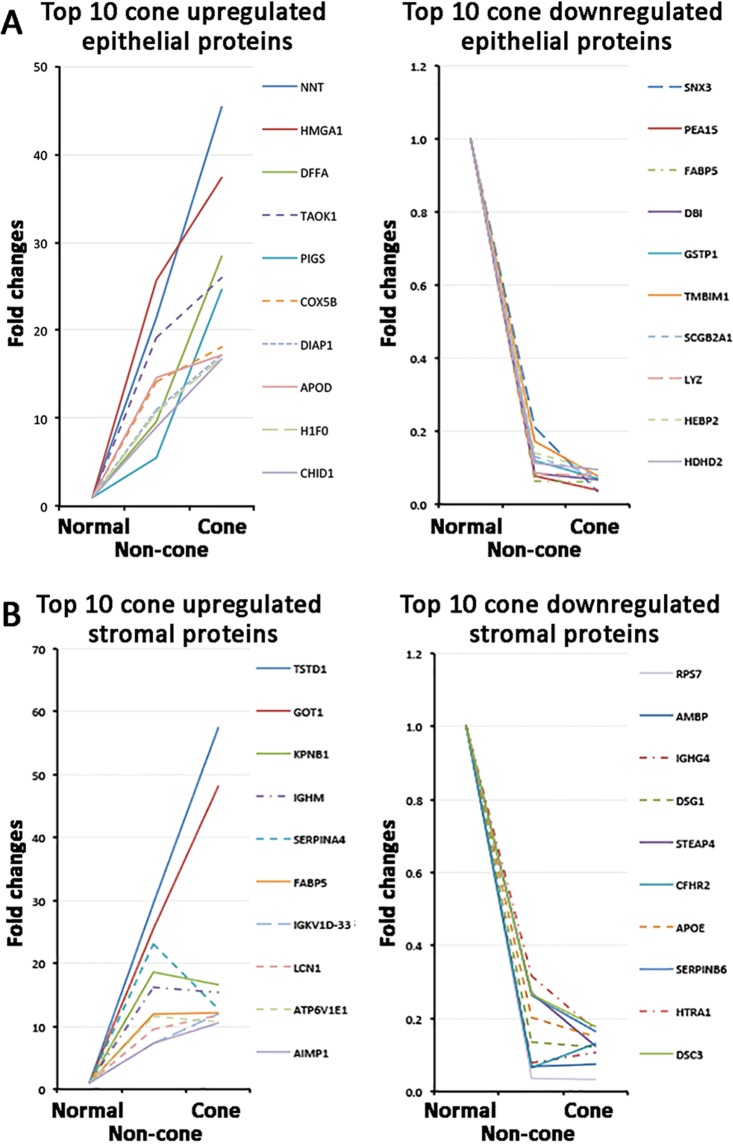
Spectral changes of top 10 differentially regulated cone epithelial (**A**) and stromal proteins (**B**) from normal to non-cone to cone stages.

**Figure 6:**
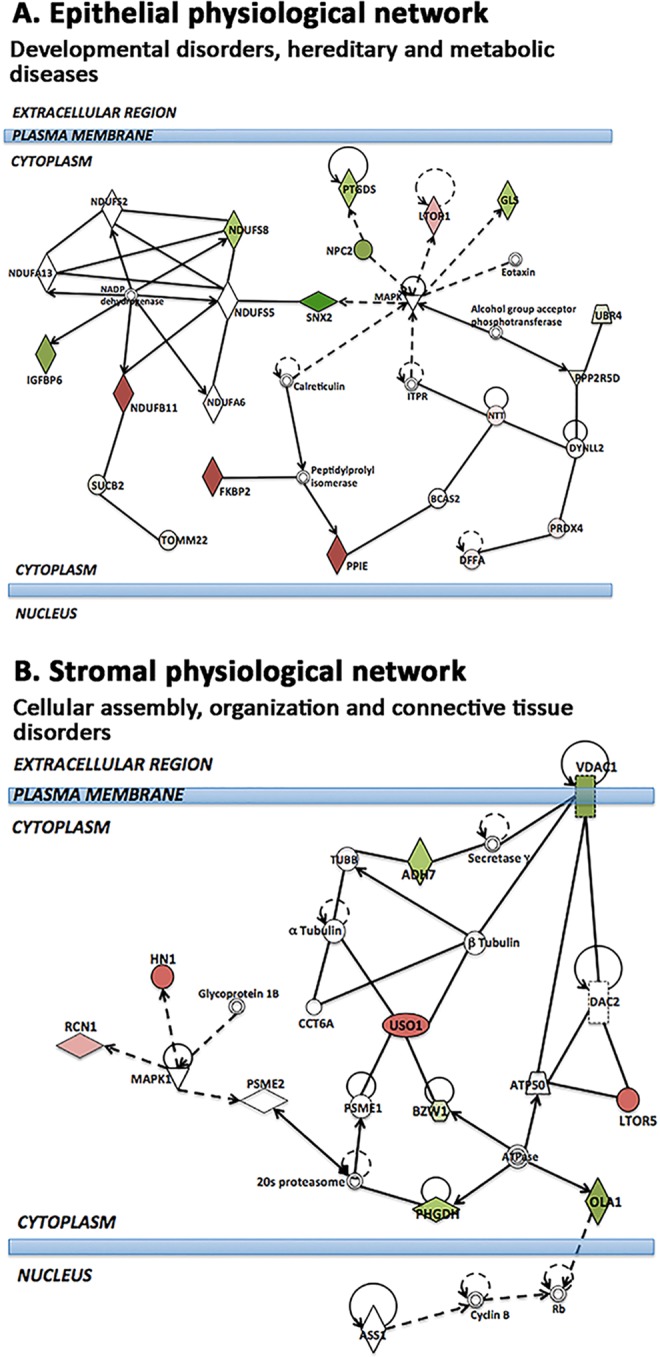
IPA interactive networks identified between KC cone and non-cone comparison. (**A**) Epithelial-derived interactome: developmental disorders, hereditary and metabolic diseases. This network shows the epithelial dysfunction due to MAPK signalling-associated changes in cell metabolism (reduced mitochondrial GLS and cell growth-related PPP2R5D, UBR4, IGFBP6, SNX2). The up-regulated PRDX4, NDUFB11 and FKBP2 suggest potential immune-related changes. (**B**) Stromal-derived interactome: cellular assembly, tissue organization and connective tissue disorders. The up-regulated microtubule protein HN1, vesicle transporter USO1 and LTOR5 as well as calcium binding RCN1, and the downregulated voltage channel VDAC1 on cell membrane and cell survival-associated ADH7, BZW1 (histone regulation), PHGDH (electron transfer in ATP synthesis) and calcium responsive OLA1 suggest perturbation of cell survival and interstitial ECM. Each network displays the genes/gene products as nodes (different shapes representing the functional classes of gene products) and the biological relationships between the nodes as lines. The length of each line reflects the amount of literature evidence supporting this node-to-node relationship. The color intensity of each node indicates the degree of upregulation (red) or downregulation (green) of the respective gene transcript. Genes in white shape are predictive to interact with the colored gene products that appear in this scheme.

Quantitative protein analysis in separated KC cone and non-cone tissues allowed the spectral examination of selected proteins along the pathogenic development from non-cone to cone stage. In cone epithelial proteome, we selected the top 10 significantly up-regulated proteins compared to normal corneal epithelium, and examined their relative expression in non-cone epithelia. Figure 4A showed these proteins possessed similar trend of elevated expression from normal, through non-cone, to the cone stage. Among them, NNT, DFFA and PIGS showed more expeditious increase (>2 folds) in cone versus non-cone than in non-cone versus normal samples. In contrast, all cone downregulated epithelial proteins were detected low in non-cone samples (Fig. 5B). In view of the differentially regulated expression, a detailed comparison of KC cone versus non-cone epithelial proteomes was conducted. Consistently found in all 4 KC pairs, there were 14 upregulated (>2 folds) and 28 downregulated proteins (<0.5 fold) (Table 1; Fig. 3). Among them, 5 were significantly upregulated (NDUFB11, GAA, DFFA, RPL28, NNT) and 15 significantly downregulated in cone epithelia (VMA5A, IGFBP6, PPP2R5D, SNX3, EIF3G, FH, NPC2, STX4, GLS, PFDN4, ARFIP1, PAFAH1B1, TOMM22, EIF2S2, ADA10) (*p* < 0.05). These proteins were submitted to the function annotation tool of DAVID bioinformatics, and only one significant enriched GO term was recognized: GO:0005739-mitochondrion (*p* = 0.048, Benjamini test; enrichment score EnS: 2.4) (Table 2). In addition, IPA identified one biochemical interactive network: developmental disorders, hereditary and metabolic diseases that could associate with the altered protein expression between KC cone and non-cone epithelia (Fig. 6A).

**Table 1:** Differentially expressed KC epithelial protein from pairwise cone versus non-cone comparison.

	UniPro Accession No.	Protein Symbol	Protein name	Mean fold changes	SD	CV	*p*
**(** ***A*** **)** ***Upregulated epithelial proteins***							
1	Q9NX14	NDUFB11	NADH:Ubiquinone oxidoreductase subunit B11	24.26	17.06	1.50	0.015*
2	P62854	RS26	Ribosomal protein S26	20.34	28.34	1.61	0.382
3	Q9NR31	SAR1A	Secretion associated Ras related GTPase 1A	18.73	15.85	1.07	0.340
4	P26885	FKBP2	FK506 binding protein 2	12.35	12.28	1.21	0.506
5	P07477	PRSS1	Protease, serine 1	11.4	20.13	1.95	0.349
6	Q6IAA8	LTOR1	Late endosomal/lysosomal adaptor, MAPK And MTOR activator 1	9.04	14.59	1.72	0.121
7	Q9Y3E1	HDGR3	Hepatoma-derived growth factor, related protein 3	8.57	13.00	1.70	0.247
8	Q9UNP9	PPIE	Peptidylprolyl isomerase E	7.69	6.32	1.02	0.146
9	P10253	GAA	Glucosidase α, acid	6.67	4.76	1.09	0.022*
10	Q96S52	PIGS	Phosphatidylinositol glycan anchor biosynthesis class S	4.37	3.23	0.90	0.127
11	Q13162	PRDX4	Peroxiredoxin 4	4.32	3.83	1.03	0.154
12	O00273	DFFA	DNA fragmentation factor subunit α	3.09	1.68	0.58	0.034*
13	P46779	RPL28	Ribosomal protein L28	3.08	1.47	0.55	0.036*
14	Q13423	NNT	Nicotinamide nucleotide transhydrogenase	2.57	1.22	0.51	0.037*
**(** ***B*** **)** ***Downregulated epithelial proteins***							
1	O00534	VMA5A	Von Willebrand factor A domain containing 5A	0.2	0.02	1.00	0.020*
2	O60493	SNX3	Sorting nexin 3	0.24	0.12	0.64	0.007*
3	P07954	FH	Fumarate hydratase	0.24	0.11	0.42	0.002*
4	Q14738	PPP2R5D	Protein phosphatase 2 regulatory subunit B′delta	0.24	0.37	1.14	0.050*
5	Q5T4S7	UBR4	Ubiquitin protein ligase E3 component N-Recognin 4	0.25	0.18	1.87	0.304
6	P24592	IGFBP6	Insulin-like growth factor binding protein 6	0.28	0.03	1.08	0.033*
7	O75821	EIF3G	Eukaryotic translation initiation factor 3G	0.3	0.2	0.73	0.013*
8	O94925	GLS	Glutaminase	0.31	0.13	0.55	0.019*
9	O00217	NDUS8	NADH:Ubiquinone oxido reductase core subunit S8	0.34	0.13	0.99	0.091
10	P23800	LOX	Lysyl oxidase	0.37	0.29	1.51	0.321
11	P14324	FPPS	Farnesyl diphosphate synthase	0.37	0.24	0.6	0.153
12	P43487	RANG	RAN binding protein 1	0.37	0.35	1.11	0.383
13	P61916	NPC2	NPC intracellular cholesterol transporter 2	0.38	0.12	0.94	0.023*
14	Q9NQP4	PFDN4	Prefoldin subunit 4	0.39	0.12	0.41	0.006*
15	Q12846	STX4	Syntaxin 4	0.41	0.24	0.61	0.027*
16	P07204	TRBM	Thrombomodulin	0.41	0.19	0.63	0.082
17	O60936	NOL3	Nucleolar protein 3	0.43	0.39	0.75	0.066
18	P53367	ARFIP1	ADP ribosylation factor interacting protein 1	0.43	0.17	0.37	0.015*
19	P43034	PAFAH1B1	Platelet activating factor acetylhydrolase 1b regulatory subunit 1	0.44	0.09	0.18	0.001*
20	O15305	PMM2	Phosphomannomutase 2	0.45	0.33	0.66	0.067
21	P20042	EIF2S2	Eukaryotic translation initiation factor 2β	0.45	0.17	0.35	0.015*
22	P41222	PTGDS	Prostaglandin D2 synthase	0.45	0.42	0.96	0.195
23	Q96H20	SNF8	SNF8, ESCRT-II complex subunit	0.46	0.46	0.84	0.177
24	Q96I99	SUCB2	Succinate-CoA ligase GDP-forming β	0.47	0.42	0.89	0.066
25	P01033	TIMP1	TIMP1 metallopeptidase inhibitor 1	0.48	0.32	0.59	0.052
26	O14672	ADAM10	ADAM metallo peptidase domain 10	0.49	0.06	0.12	0.000*
27	Q9NS69	TOMM22	Translocase of outer mitochondrial membrane 22	0.49	0.21	0.38	0.015*
28	P46782	RS5	Ribosomal protein S5	0.49	0.36	0.86	0.054

These proteins had same changes in all 4 KC sample pairs. Note: Mean fold changes from all 4 KC sample pairs; SD – standard deviation; CV: coefficient of variation; *p*: significance value (paired Student’s t-test; *p < 0.05 denotes statistically significance).

**Table 2:** Enriched Gene Ontology terms and KEGG pathways identified for differentially expressed proteins between KC cone and non-cone epithelial samples.

	Enrichment score	Biological events	Proteins (UniPro Accession no.)	*p*
**(** ***A*** **)** ***Enriched GO terms*** **(** ***DAVID*** **)**				
1	2.36	GO:0005739 ~ mitochondrion	P14324, Q9NS69, O94925, Q9NX14, Q96I99, P07954, Q9NQP4, O00217, O60936, Q13162, Q13423	0.048*
2	1.36	GO:0003723 ~ RNA binding	Q9UNP9, P46779, O75821, O60936, P46782, P20042	0.479
**(** ***B*** **)** ***KEGG pathways*** **(** ***DAVID*** **)**				
1	2.36	hsa01100: Metabolic pathways	O94925, Q9NX14, P41222, P43034, O00217, P14324, P54819, P10253, Q96I99, P07954, O15305, Q13126, Q13423	0.321

The biological events were ranked using enrichment scores. *Adjusted Benjamini *p* < 0.05 represents statistical significance.

### Stromal proteomes

#### KC cone versus normal corneal stromal tissue

A list of 108 proteins were significantly upregulated (*p* < 0.05) and 110 significantly downregulated in all 4 KC cone samples, when compared to normal corneal stroma (Fig. 3; Supplemental Data File S2C; raw data in Supplemental Data File S1). They included reported proteins like Col1A2, MMP9 and SOD. Heat map showed distinct dendrographic clustering in cone versus normal stromal proteomes (Fig. 4A). Using DAVID, these proteins were associated to significant enriched GO terms: cell-cell adhesion (*p* < 0.001; EnS:10.7), extracellular region (*p* < 0.001, EnS:7.9), protein folding (*p* < 0.001, EnS:5.4), classical complement pathway (*p* < 0.001, EnS 5.1), Fc ε receptor signalling pathway (*p* < 0.001, EnS:5.1), GTP binding (*p* = 0.035, EnS:3), intermediate filament (*p* = 0.011, EnS:2.8), negative regulation of endopeptidase activity (*p* < 0.001, EnS:2.7) and response to ROS (*p* = 0.012, EnS:2.4) (Supplemental Table S3). It also revealed 2 significant KEGG pathways: hsa04610: complement and coagulation cascades (*p* = 0.006, EnS:2.9) and hsa00010:glycolysis/gluconeogenesis (*p* = 0.046, EnS:2). IPA showed biological pathways including LXR/RXR activation (*p* < 0.001), acute phase response signalling (*p* < 0.001), complement system (*p* < 0.001), FXR/RXR activation (*p* < 0.001) and glycolysis (*p* < 0.001). In the physiological and disease category, the KC cone stromal proteome affected cell death and survival, dermatological disease and conditions, inflammatory disease and response.

#### KC non-cone versus normal corneal stromal tissue

We detected 130 proteins significantly upregulated (*p* < 0.05) and 86 significantly downregulated in all 4 KC non-cone proteomes compared to that of normal corneal stroma (Fig. 3; Supplemental Data File S2D). There was no clear clustering between non-cone and normal stromal proteomes as revealed by heat map analysis (Fig. 4B). The function annotation of DAVID showed these proteins were related to significant enriched GO terms, namely cell-cell adhesion (*p* < 0.001, EnS:9.3), classical complement activation pathway (*p* < 0.001, EnS:6.5), Fc γ-receptor signalling pathway involved in phagocytosis (*p* < 0.001, EnS:6.5), immune response (*p* = 0.01, EnS:6.5), extracellular region (*p* < 0.001, EnS:5.6), oxidoreductase activity (*p* < 0.001, EnS:3.4), gluconeogenesis (*p* = 0.016, EnS:3.4), immunoglobulin receptor binding (*p* = 0.001, EnS:3.1), unfolded protein binding (*p* = 0.007, EnS:2.9), protease binding (*p* = 0.021, EnS:2.8) and collagen catabolic process (*p* = 0.003, EnS:2.4) (Supplemental Table S4). Two significant KEGG pathways were noted - hsa00010: glycolysis/gluconeogenesis (*p* = 0.007; EnS:2.5) and hsa04512:ECM-receptor interaction (*p* = 0.023; ErS:2.4) (Supplemental Table S4). Similar biological events were revealed using IPA and the top 5 canonical pathways were LXR/RXR activation, FXR/RXR activation, acute phase response signalling, complement system and glycolysis. The affected physiological and functional category were (1) cell-to-cell signalling; (2) inflammatory response and abnormalities (3) haematological disorders; (4) dermatological diseases, inflammatory disorders and response and (5) tissue infection and inflammation.

#### Differential expression of stromal proteins in KC cone versus non-cone

The top 10 significantly up- and down-regulated cone stromal proteins compared to normal stroma were also altered in non-cone samples. We observed 2 cone upregulated proteins (TSTD1, GOT1) had a nearly linear elevation from normal, through non-cone, to the cone stage, but the fold changes were <2 (Fig. 5B). Others had minor fluctuations between cone and non-cone samples. On the other hand, all cone downregulated stromal proteins were also detected low in non-cone samples (Fig. 5B). When KC cone and non-cone stromal proteomes were compared in pair-wise manner, there were 14 upregulated and 22 downregulated proteins in all 4 pairs of KC pairs (Table 3; Fig. 3). These included the KC reported protein, LOX, which was more suppressed in cone than non-cone stroma (0.5 ± 0.2 fold). Two proteins (SYTC, RUVBL2) were significantly upregulated and 12 were significantly downregulated (TACSTD2, VIT, STEAP4, SAR1A, PHDGH, ABCB11, SF3B3, H2AJ, MIF, NQO1, BZW1, ADH7) in cone stroma. By DAVID function annotation, the proteome changes between cone and non-cone stroma did not associate with any significant enriched GO terms (Table 4). However, one KEGG pathway was significantly noted (hsa04141: protein processing in endoplasmic reticulum, *p* = 0.008, Benjamini test; enrichment score EnS:1.8). IPA identified one putative biochemical network: cellular assembly, tissue organization and connective tissue disorders (Fig. 6B).

**Table 3:** Differentially expressed KC stromal protein from pairwise cone versus non-cone comparison.

	UniPro Accession No.	Protein Symbol	Protein name	Mean fold changes	SD	CV	*p*
**(** ***A*** **)** ***Upregulated stromal proteins***							
1	P26639	SYTC	Threonyl-tRNA synthetase	12.22	6.32	2.75	0.021*
2	Q9Y230	RUVBL2	RuvB like AAA ATPase 2	11.56	5.18	1.75	0.025*
3	P52597	HNRPF	Heterogeneous nuclear ribonucleoprotein F	8.72	8.77	1.01	0.156
4	Q15293	RCN1	Reticulocalbin 1	8.38	5.82	0.92	0.185
5	Q04828	AK1C1	Aldo-keto reductase family 1 member C1	7.79	5.32	1.63	0.214
6	O43504	LTOR5	Late endosomal/lysosomal adaptor, MAPK And MTOR activator 5	6.71	4.22	1.08	0.130
7	P27169	PON1	Paraoxonase 1	4.94	3.81	0.77	0.230
8	P01714	IGLV3-19	Immunoglobulin lambda variable 3-19	4.83	7.06	1.46	0.250
9	O43396	TXNL1	Thioredoxin like 1	4.29	2.85	0.88	0.292
10	Q9UK76	HN1	Hematological and neurological expressed 1	3.75	3.28	0.99	0.109
11	P01602	IGKV1-5	Immunoglobulin kappa variable 1–5	3.17	2.03	0.64	0.056
12	P0CG06	LAC3	Immunoglobulin lambda constant 3 (Kern-Oz + Marker)	3.02	2.17	0.79	0.091
13	O60763	USO1	USO1 vesicle transport factor	2.84	2.33	0.82	0.093
14	P14780	MMP9	Matrix metalloproteinase 9	2.38	0.97	0.41	0.251
**(** ***B*** **)** ***Downregulated stromal proteins***							
1	P09758	TACSTD2	Tumor-associated calcium signal transducer 2	0.32	0.14	0.55	0.006*
2	P34896	SHMT1	Serine hydroxymethyl transferase 1	0.32	0.31	0.98	0.088
3	Q9NTK5	OLA1	Obg like ATPase 1	0.32	0.28	0.88	0.053
4	Q9H0W9	C11orf54	Chromosome 11 open reading frame 54	0.34	0.32	0.93	0.092
5	Q6UXI7	VIT	Vitrin	0.35	0.09	0.55	0.047*
6	P11166	SLC2A1	Solute carrier family 2 member 1	0.39	0.42	1.06	0.131
7	O95342	ABCB11	ATP binding cassette subfamily B11	0.4	0.14	0.36	0.017*
8	Q9NR31	SAR1A	Secretion associated Ras related GTPase 1A	0.4	0.23	0.58	0.046*
9	P15559	NQO1	NAD(P)H quinone dehydrogenase 1	0.42	0.14	0.50	0.021*
10	Q9BTM1	H2AJ	H2A histone family member J	0.42	0.15	0.35	0.016*
11	Q687X5	STEAP4	STEAP4 metalloreductase	0.44	0.12	0.74	0.041*
12	P14174	MIF	Macrophage migration inhibitory factor	0.44	0.18	0.41	0.025*
13	Q15393	SF3B3	Splicing Factor 3b Subunit 3	0.44	0.25	0.56	0.042*
14	P18510	IL1RN	Interleukin 1 receptor agonist	0.44	0.36	0.81	0.068
15	P62318	SMD3	Small nuclear ribonucleoprotein D3 polypeptide	0.46	0.37	0.82	0.171
16	O43175	PHGDH	Phosphoglycerate dehydrogenase	0.47	0.25	0.70	0.035*
17	Q7L1Q6	BZW1	Basic leucine zipper and W2 domain 1	0.47	0.23	0.48	0.047*
18	Q9BTV4	TMM43	Transmembrane protein 43	0.48	0.35	0.73	0.091
19	P55769	NH2L1	SNU13 homolog, small nuclear ribonucleoprotein (U4/U6.U5)	0.48	0.34	0.59	0.086
20	P40394	ADH7	Alcohol dehydrogenase 7 (Class IV), Mu Or Sigma polypeptide	0.49	0.01	0.35	0.016*
21	P23800	LOX	Lysyl oxidase	0.49	0.24	1.88	0.276
22	P21796	VDAC1	Voltage dependent anion channel 1	0.49	0.36	0.72	0.217

These proteins had same changes in all 4 KC sample pairs. Note: Mean fold changes from all 4 KC sample pairs; SD – standard deviation; CV: coefficient of variation; *p*: significance value (paired Student’s t-test; *p < 0.05 denotes statistical significance).

**Table 4:** Enriched Gene Ontology terms and KEGG pathways identified for differentially expressed stromal proteins in KC cone and non-cone comparison.

	Enrichment score	GO terms	Proteins (UniPro Accession no.)	*p*
**(** ***A*** **)** ***Enriched GO terms*** **(** ***DAVID*** **)**				
1	2.26	GO:0098609 ~ cell-cell adhesion	O60763, Q7L1Q6, P61026, P09758, P14618, Q9NTK5	0.342
2	1.85	GO:0055114 ~ Oxidation-reduction	O43396, P40394, Q687X5, P15559, O43175, Q04828	0.785
3	1.73	GO:0005739 ~ Mitochondrion	O43396, P34896, P51572, P14618, P50213, P21796	0.956
4	1.34	GO:0005525 ~ GTP binding	Q9NR31, Q9NVJ2, P61026, Q9NTK5, P61224	0.454
5	1.2	GO:0000398 ~ mRNA splicing, via spliceosome	P52597, P55769, P62318, Q15393	0.816
6	1.15	GO:0038096 ~ Fc-gamma receptor signaling pathway involved in phagocytosis	O15145, P01714, P01602, P0CG06	0.676
7	1.15	GO:0038095 ~ Fc-epsilon receptor signaling pathway	P62837, P01714, P01602, P0CG06	0.848
8	1.15	GO:0006958 ~ Complement activation, classical pathway	P01714, P01602, P0CG06	0.759
9	1.04	GO:0006886 ~ Intracellular protein transport	Q9NR31, O60763, Q9UNH7, P51572	0.774
**(** ***B*** **)** ***KEGG pathways*** **(** ***DAVID*** **)**				
1	1.77	hsa04141: Protein processing in endoplasmic reticulum	Q9NR31, P62837, P14314, P39656, P04843, P51572, P05198	0.008*
2	1.73	hsa01100: Metabolic pathways	P34896, P14618, P50213, O43175	0.331
3	1.2	hsa03040: Spliceosomes	P55769, P62318, Q15393	0.883

The pathways were ranked using enrichment scores. *Adjusted Benjamini *p* < 0.05 represents statistical significance.

### Tissue expression of selected proteins

**Figure 7:**
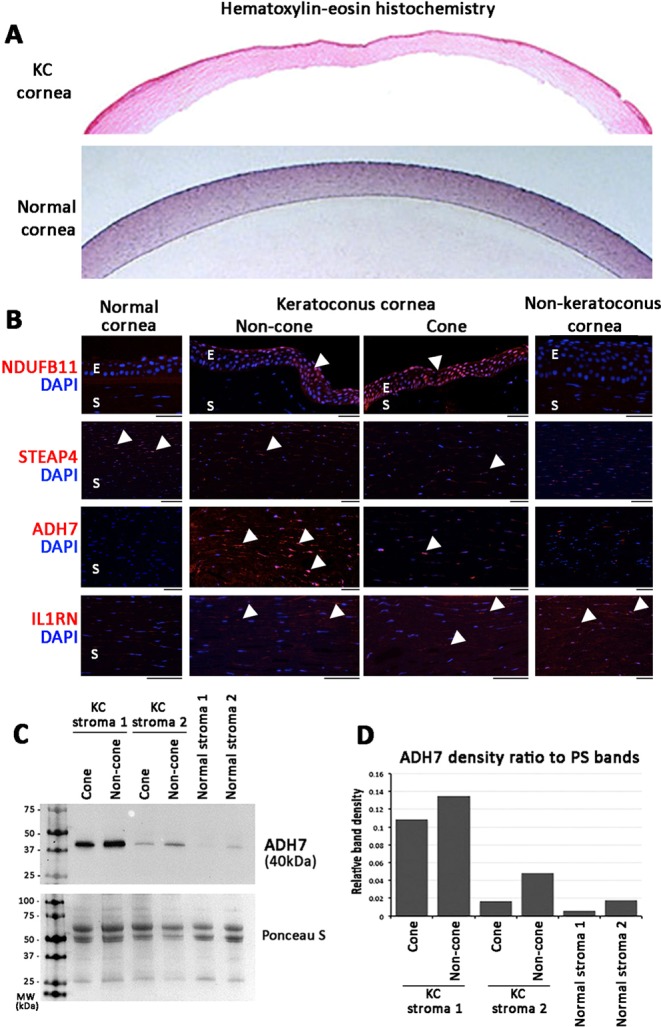
Immunohistochemistry showing the tissue expression of selected proteins in archived normal, KC and non-KC corneal specimens. (**A**) Hematoxylin-eosin images of KC and normal corneas. (**B**) Strong nuclear signal (arrowheads) of NDUFB11 was detected in KC epithelia (labeled E) but faint in non-KC and normal cornea. STEAP4 metalloproteinase was faintly stained in KC stroma (labelled S) than in normal corneal stroma. ADH7 was clearly detected in KC non-cone stroma (arrowheads), and was less in cone stroma. Negligible expression was found in normal and non-KC corneal stroma. IL1RN was mildly expressed in KC and non-KC stroma but faint in normal cornea. (**C**) Western blotting of ADH7 in KC and normal stromal fractions. (**D**) Band densitometry showed ADH7 was upregulated in KC non-cone than in cone stromal fractions. Normal corneal stromal samples had reduced expression. Full-length gel images are shown in Supplemental Fig. S4.

We selected 4 proteins for validation study using a different cohort of 4 advanced KC corneas compared to 4 non-KC samples and 2 normal corneal samples (Supplemental Table S5, representative hematoxylin-eosin pictures in Fig. 7A). These proteins were chosen based on their involvement in mitochondria (for NDUFB11), protein trafficking (for STEAP4), immune and inflammatory response (for IL1RN), and response to oxidative stress (for ADH7) (more explanation in Discussion) as well as the availability of high quality antibodies for immunohistochemistry on paraffin sections (Supplemental Table S6).

The KC-upregulated NDUFB11 (NADH:ubiquinone oxidoreductase subunit B11; UniPro Q9NX14) was positively stained in corneal epithelia in 3 of 4 KC specimens and was negative in normal cornea (0/2) and non-KC samples (0/4) (Fig. 7B). Similarly, ADH7 (alcohol dehydrogenase 7; P40394) was positively stained in KC samples (4/4), particularly in non-cone region and negligibly in non-KC and normal corneas (Fig. 7B). This was consistent to the proteomic data (KC cone/normal: 6.5 ± 2.6 folds; KC non-cone/normal: 11.4 ± 6.3 folds; KC cone/non-cone: 0.49 ± 0.08 fold, *p* = 0.016) (Table 3). Using western blotting, ADH7 was revealed at ~40 kDa position and expressed stronger in non-cone stromal samples than in cone samples (Fig. 7C). Analyzed by band densitometry, ADH7 expression was elevated in KC non-cone by 27% to 2.8 folds, when compared to the corresponding cone stroma (Fig. 7D). On the contrary, STEAP4 metalloproteinase (UniPro Q687X5) was faintly stained in KC stroma (1/4) than in normal corneal stroma (2/2) (Fig. 7B). This result matched to the stromal proteomic data that STEAP4 was suppressed in KC cone and non-cone stroma (Table 3). IL1RN (Interleukin 1 receptor agonist; P18510) was detected in KC cone and non-cone stroma as well as in non-KC samples, however the normal corneal stroma showed negligible staining (Fig. 7B).

## Discussion

This study investigated the epithelial and stromal proteomes of 2 topographically divergent regions of KC cornea, i.e. cone with thin ectatic topography and non-cone with normal topography, using label-free SWATH-MS. This is the first proteome study that compared variations in cone and non-cone tissues of human KC corneas. Previous studies have examined whole KC epithelial or stromal tissue in comparison to normal corneas to obtain KC-associated expression patterns. However, little is known about the biological variations in these 2 regions and whether the pathological biological changes are restricted to the ectatically abnormal cone or extends to the rest of cornea. In this study, we reported proteins with expression changes (in the range of ≥2 folds or ≤0.5 fold) consistently found in all 4 KC samples. Our results showed that the proteome changes were not limited to the topographically thinner and mechanically weakened cone region but also in the non-cone part, indicating a peripheral involvement in the pathogenic KC development. Using DAVID functional annotation to identify enriched GO terms and IPA to depict biochemical interactive networks, the epithelial proteome change between cone and non-cone was related to cell metabolism with mitochondrial involvement, whereas the stromal alterations were linked to cellular assembly and tissue organization. Using immunohistochemistry and western blotting, four selected proteins with reported activities on mitochondria, oxidative stress, immune and inflammatory responses were validated to follow the same trend of changes as seen in our proteomic study.

KC pathogenesis has been known to be complex and a variety of cellular and extracellular processes are involved in the corneal ectatic changes and focal thinning^1,11^. In the past, KC was defined as a non-inflammatory corneal degenerative disorder due to the lack of cellular infiltration and neovascularization^10^. However, increasing evidence has contradicted this theory and implicated local inflammatory and immune responses as well as oxidative stress as the potential triggering factors^29,30,35,41^. This is consistent with our data, which showed the involvement of immune pathways in both cone and non-cone stroma, such as Fc γ- and ε-receptor signalling as well as complement cascade. In addition, the enzymatic imbalance, including the upregulation of MMPs and reduced LOX activity, affects collagen polymerization and its crosslinking with elastin fibers^52,53^. The disorientation of stromal collagen pattern, in addition to the metabolic changes, altered cell-cell interactions and cell death, would reduce the biomechanical strength of stroma and its stability. Measurement of stress-strain curves showed a reduced elastic modulus in advanced KC corneas^54^. This biomechanical weakening will drive the topographical changes of focal distortion and thinning^55^.

The topographic deformation is localized to the cone region. Using Brillouin frequency shift microscopy, the cone region showed mechanically weakening with a lower elastic modulus, while the peripheral non-cone had similar modulus to the normal corneas^56^. However, this biomechanical data did not show if the biological changes are restricted to the cone or extended to the rest of cornea. Our data has located target protein changes in cone stroma, including the reduction of Col1A1 and 2 (both ~0.3 fold as control), Col6A1 to 3, DSC3, FMOD, BGN, TSP4 and TIMPs, and increase of serpin family members (A4, A5, F2) and MMPs, which were indicative of stromal matrix dysregulation (Supplemental Data File S2C). This would affect collagen fibril orientation and interweaving and increased fibril degradation resulting in loss of stromal matrix stabilization and stromal mass^57^. In contrast, the expression of Col1A1 and 2 (both ~0.5 fold as control), BGN and TIMP2 were less reduced in non-cone stroma. This might cause insignificant stromal alterations and thinning in non-cone region^55,56^.

Our data also showed the upregulation of various immune-related regulators and mediators in cone stroma (such as IGKV1D-33, 2D-28, IL18, C2, C4A, C9, CFD, CFH, TF, TOLIP) and this predicted the classical and alternative complement cascades, which could activate collagenases and metalloproteinases^29,31^ and trigger the prostaglandin pathway (PTGDS and PTGS2) in inflammatory responses. Cell metabolic dysregulation in cone stroma was suggested by the altered expression of ribosomal proteins (RPS7, RPL11, RPS21, RLAO), IGFBP, ASPH, YWHAZ, histone family (HIST2H2BE, H3F3A) and interleukin 1 receptor antagonist IL1RN (Supplemental Data File S2C). Our validation study using archived KC samples showed that IL1RN was detected in KC cone and non-cone stroma but negligible in normal stroma by immunohistochemistry (Fig. 7B). This IL1 cytokine family member regulates the activities of IL1α and β and modulates IL1-related immune and inflammatory responses. Earlier report by Nowak *et al*. had shown a substitution c.214 + 242C > T of IL1RN in an Ecuadorian KC family and proposed the altered interaction between IL1 and its cell surface receptor in KC etiology^58^. Moreover, downregulation of retinol binding protein RBP3, integrin ITGA6 and lamin LMNB would suppress cell growth and cell-cell interactions among stromal keratocytes resulting in reduced stromal homeostasis.

This differential protein expression was further inspected by the spectral changes for the top 10 cone upregulated stromal proteins in non-cone region (Fig. 5B). We observed 2 types of changes. Type 1 with linear elevation from normal via non-cone to cone stage was found for TSTD1 and GOT1, which were the top upregulated proteins in cone stroma. TSTD1 (thiosulfate sulfurtransferase like domain containing 1) is a mitochondrial complex I enzyme for ATP production under hypoxia condition^59^ and relates to prostaglandin metabolism in oxidative stress-mediated cell death^60^. GOT1 is also a mitochondrial enzyme for ATP production via tricarboxylic acid cycle. Using L-cysteine as substrate, it regulates the level of mercaptopyruvate in producing hydrogen sulfide, which acts as a synaptic modulator^61^ and a substrate of sulfurtransferase TSTD1. Whether this relates to the cell metabolic changes and loss of corneal sensitivity in KC cornea is yet to be illustrated. The remaining proteins belonged to Type 2, which had elevation from normal to non-cone but stable or decrease in cone stage. SERPINA4 (serine proteinase inhibitor 4, kallistatin), an inflammation and oxidative stress mediator, has been found to suppress oxidative stress-associated NAD(P)H oxidase expression^62^. IGHM, IGKV1D-33 and LCN1 are related to inflammatory responses; ATP6V1E1 and FABP5 for mitochondrial ATP production; and AIMP1, KPNB1 for cellular metabolism.

Proteins that were altered in non-cone stromal proteome (compared to normal) were involved in similar biological pathways as in cone stroma, such as cell-cell adhesion, complement cascade, immune responses and oxidoreductase activity (Supplemental Table S4). This illustrated that considerable biological events have occurred in non-cone stroma even though it is topographically similar to normal. Since many stromal proteins are structural and their turnover is slow^63^, the pathological changes may remain undetectable until additional triggering factors are combined. This could be related to protein folding, Fc receptor activation, ROS responses and collagen degradation, which were identified for cone stromal proteome changes (Supplemental Table S3). Activation of Fc γ and ε-receptors could trigger the release of potent inflammatory mediators, which stimulate proteases^30,64,65^. Increased cell death due to oxidative stress-related protein misfolding and mistrafficking as well as collagen degradation would lead to the stromal matrix degeneration as a major KC process. This was validated when we compared KC cone and non-cone stroma in a pairwise manner. IPA identified the cellular assembly and tissue organization as the altered functional pathway. The altered protein expression in all 4 KC samples underscored cytoskeletal regulation, such as the increased microtubule protein HN1, vesicle transporter USO1 and LTOR5 as well as calcium binding RCN1, and the downregulated voltage channel VDAC1 on cell membrane and cell survival-associated STEAP4 (mitochondrial), ADH7 (defence against oxidative stress), BZW1 (histone H4 regulation), PHGDH (electron transfer in ATP synthesis) and calcium responsive OLA1 (Supplemental Table S7). The results were validated using immunohistochemistry and western blotting. Stronger ADH7 expression was found in KC non-cone than in cone stroma. Besides its anti-oxidation activity, ADH7 participates in retinoic acid (RA) synthesis and retinoid X receptor (RXR) signalling^66^ that could regulate stromal cell survival and matrix homeostasis. Loss of RA synthesis has been shown to reduce corneal stromal thickness and increase apoptosis, resulting in corneal thinning^67^. Additionally, the suppressed STEAP4 metalloproteinase could indicate mitochondrial damage and oxidative stress, affecting stromal keratocyte function and viability^68^. We also demonstrated that while some proteins followed linear expression change from normal over non-cone to cone stage, others did not. Hence, in addition to the theory of stromal degeneration in association to inflammation, immune deregulation, oxidative stress and cell death in KC development^25,44–47^, our data provided new insights showing that stromal cell metabolic changes, viability and collagen degradation would contribute to the focal degeneration and topographic deformation in the cone stromal region.

Though many researchers have proposed that abnormal stromal metabolism is the primary site of corneal dysfunction in KC, various histopathological studies have highlighted the epithelial abnormalities and postulated that insults to the epithelium cause a release of proteolytic enzymes (such as MMP9, IL6 and LOX) to degrade the stromal collagen and matrix components^1,53,69,70^. *In vivo* confocal imaging studies further described that the corneal epithelium was affected in correlation to the KC severity^71–73^. Altered gene expression related to Wnt, Hedgehog and Notch signalling in the entire KC epithelium was also demonstrated by a recent transcriptomic study^47^. However the initial changes of epithelium in KC progression and in the 2 topographically different regions remain unclear. Our proteome analysis had revealed altered protein expression in KC non-cone epithelium and was related to cell-cell adhesion, mitochondrial electron transport, ATP synthesis, ROS responses and cytoskeleton. Hence, considerable biological changes have occurred in the non-cone epithelium. Additional changes of RNA metabolism in the cone epithelium are linked to the altered protein synthesis, cellular stress and cell death. Cell stress-related expression of cytokines and mediators could activate proteolytic enzymes to degrade collagen matrix and weaken the cornea. Abnormal autophagy and apoptosis as a consequence of oxidative stress has been reported in KC epithelia^74^, leading to the reduction of epithelial cell number and thinner apical epithelium. When comparing between KC cone and non-cone epithelial proteomes, the changes were associated to cell metabolism as the enriched GO term and mitochondria as the KEGG pathway (Table 2). This was supported by IPA physiological network, which showed developmental disorders and metabolic diseases. In addition, the altered epithelial proteome changes encoded cell growth and immune response, including LTOR1 in MAPK signalling; cell survival-related IGFBP6, PPP2R5D, SUCB2 (ATP synthesis); mitochondrial NDUFS8, NDUFB11, GLS and TOMM22; PPIE, SNX2, FKBP2 in protein folding and vesicle trafficking; UBR4 in protein degradation; and immune-related PRDX4, NPC2 (Supplemental Table S7). We also validated the altered expression of NDUFB11 in archived KC epithelia compared to non-KC and normal corneal epithelia. This mitochondrial inner membrane protein belongs to multisubunit NADH:ubiquinone oxidoreductase (complex 1) transferring electrons from NADH to ubiquinone of the mitochondrial respiratory chain. This altered expression between KC cone and non-cone epithelia was supported by previous finding of nonsense mutation in NDUFB11 in association to microphthalmia (with symptoms of microcornea and corneal topographic changes) with linear skin defects^75^.

In this study, the KC cone and non-cone tissues were separated by manual dissection according to the pre-operative topographic data. Intra-operatively, the cone and non-cone regions were carefully demarcated by marking on the epithelium using a surgical skin marker. This warranted a precise separation of cone and non-cone tissues under dissecting microscope. Subsequent bright-field imaging and tissue dimension showed similar differences as in the Pentacam pictures. As the cone apices were decentered inferiorly from the pupil axis, the non-cone regions were obtained from the superior portion and varied between 1/3 to 1/2 of the overall resected corneal tissue, depending on the cone location. Likewise, the inferior region, where cone was located, varied between 1/2 to 2/3 of the resected corneal tissue. These dimension ratio variations were replicated by the extracted stromal protein quantities, of which the cone fractions were often greater than non-cone fractions (Fig. 2C). In order to attain a reproducible separation of cone region from the excised cornea, topography-guided laser-assisted dissection could be designed instead of manual cutting. However, the issues of tissue volume changes (stromal edema) under *ex vivo* storage and thermal effect on protein degradation could be the major concerns.

Even though this pilot study appeared to be limited by the sample size, we were able to identify protein groups with consistent expression changes (≥2 folds or ≤0.5 fold) in all sample pairs, indicating their significant roles in KC progression. As anticipated, a small number of proteins (20 epithelial and 14 stromal) were significantly altered between cone and non-cone samples and subsequent IPA illustrated only single network affected in either region. Future study with larger sample size would improve the precision and assure these proteins to be involved in the biological changes in KC pathogenesis and to derive the potential mechanisms of action. In addition, while KC corneas were separated into cone and non-cone regions, the normal cornea was taken as a whole. Whether the central and peripheral corneal regions possess different protein composition and expression profiles, as well as whether this plausible difference influences the pathogenic development of KC cone and non-cone regions await to be confirmed.

Our study has demonstrated that the non-cone region undergoes biological and biochemical changes, even though it is topographically normal. Hence, the altered pathophysiology is not limited to the topographically thinner and mechanically weakened cone apex but involves the non-cone region as well. The variation in protein expression has provided us with more information to understand the disease mechanism and progression of KC. Future work focusing on these early biochemical changes may elucidate new biomarkers for early KC in topographically normal eyes.

## Methods

### Human corneal samples

Four KC Asian patients with mean age 25.3 ± 5.1 years (range, 20–32) and gender 1:1 (male:female) (Fig. 2A) were recruited at the Singapore National Eye Centre. The study was performed in accordance with the Declaration of Helsinki and approved by SingHealth Centralized Institutional Review Board (CIRB 2016/2141). Written informed consents were obtained from all participants. Disease diagnosis was made by a corneal specialist (JSM). This was based on the findings on corneal topographies and clinical signs assessed with slit lamp biomicroscopy, and manifest refraction. Normal corneal epithelia were collected from two Chinese patients (aged 26 and 34) with normal corneal topographies undergoing photorefractive keratectomies for refractive error corrections. Normal corneal stroma was obtained from research grade corneal tissues (donor age: 31 and 37 years) deemed unsuitable for transplantation (clear corneas and corneal endothelial cell count >2200/mm^2^) and collected by The Lions Eye Institute for Transplant and Research Inc. (Tampa, FL, USA), following institutional review board approval. Consent was taken at the time of retrieval by the next of kin for use in research. Donor corneas were procured within 24 h after death, preserved in Optisol-GS storage medium (Bausch-Lomb, Rochester, NY, USA) and transported on ice to the laboratory. For the validation of candidate protein expression, an additional cohort of archived pathological KC samples (n = 4), corneal deep scar samples (n = 4) and normal cornea samples (n = 2) (Supplemental Table S5), were obtained from the Pathology Division, Singapore General Hospital, Singapore under valid CIRB approval. They were processed for paraffin embedding and sectioning for immunohistochemistry.

### Corneal tissue processing and protein preparation

KC corneas were immediately placed in ice-cold phosphate buffer saline (PBS, Invitrogen, Carlsbad, CA, USA) and delivered on ice to the laboratory. Cone and non-cone regions were separated under dissecting microscopy, with reference to the pre-operative topographic data (Fig. 2A,B) and the demarcation of cone and non-cone tissue marked intra-operatively on the corneal epithelium by a surgical skin marker (Medline, IL). Cone and non-cone regions were separated under dissecting microscopy, according to the pre-operative topographic data (Fig. 2D). Epithelia of both cone and non-cone tissues were carefully scraped and collected, leaving the corneal stroma intact. All samples were processed for protein extraction as described previously^25^. In brief, tissue fragments were placed in 0.5% sodium dodecylsulfate (SDS, Sigma, St Louis, MO, USA) in PBS (150 μl), homogenized by a 2 ml Biomasher (USA Scientific, Ocala, FL, USA) and high-density sonication for 30 sec on ice. After centrifugation (15,000 × g, 10 min, 4 °C), the supernatant was collected as KC cone epithelial, cone stromal, non-cone epithelial, and non-cone stromal fractions, together with normal corneal epithelial and normal corneal stromal fractions. The protein profile and quality was assessed by 4–20% gradient SDS-polyacrylamide gel electrophoresis (SDS-PAGE, BioRad, Herculus, CA, USA), followed by Coomassie brilliant blue R-250 (Sigma) visualization (Supplemental Fig. S1) and imaged with ChemiDoc system (BioRad).

### Quantitative proteomics (SWATH-MS analysis)

Protein samples (at constant 100 μg protein loads, to eliminate quantitative changes due to the corneal volume variation in the thinned cone region compared to the relatively thicker non-cone region) were trypsinized and peptides were processed for LC-MS/MS analysis and ion library data were collected by Information dependent acquisition (IDA) analysis. IDA data from four runs were combined and processed using ProteinPilot 5.0 (AB Sciex) with database search using uniprot_sprot_Sept16. Protein identification with False Discovery Rate (FDR) <1% and other parameter settings were similar as previously reported^76^. SWATH data were processed using SWATH quantitation function in Peakview 2.2. Protein quantification was calculated using 5–6 transitions per peptide and 1–10 peptides (99% peptide confidence level). The individual protein quantity was normalized to the total ion current. All procedure details were referred to Supplemental Information.

#### MS data analysis

The ratios of signal intensity of test samples and internal control were used to perform clustering analysis. The one with fluorescence intensity higher than 800 after subtracting the background was considered as an expressed signal. Clustering was done using Cluster 3 software (http://bonsai.hgc.jp/~mdehoon/software/cluster/software.htm#ctv) (clustering type: hierarchical clustering, Distance metric: Pearson correlation).

#### Differential protein expression, pathway and statistical analyses

SWATH data derived epithelial and stromal proteome lists from KC cone, non-cone and normal corneal samples. The ratios were calculated from the respective comparisons (cone versus normal; non-cone versus normal and pairwise comparison between cone and non-cone samples). We selected stringent cut-off values of ≥2 folds (upregulation) and ≤0.5 fold (downregulation) between samples^51,77^. Smaller fold changes (>0.5 fold to <2 folds) could be due to technical variations, hence the proteins may be non-representative of real changes, unable to activate certain pathways *in situ*, and unable to cause pathological effects, in particular the structural and biomechanical alterations in this KC study. The geometric means and standard deviation (SD) were obtained and T-test based on the log transformation of ratios was used to calculate *P* values. To ensure reliable data collection, we employed stringent filtering that only proteins having consistent change in all 4 KC cone and non-cone sample pairs were analyzed. Proteins with statistically significant changes (*p* < 0.05) without the concordant changes in all 4 KC pairs were excluded.

*Gene Ontology (GO) term analysis* to identify enriched biological themes was done by inputting database to web-based DAVID Bioinformatics Resources v6.8 (NIAID, NIH, USA). Adjusted Benjamini P values were calculated by Modified Fisher Exact test with the smaller P values representing higher chance of enrichment.

*Ingenuity Pathway Analysis* (*IPA, Qiagen, Hilden, Germany*) to identify the cellular distribution of proteins and association of proteins with disease, molecular and cellular functions, physiological system development and canonical pathways.

### Selected protein validation

#### Immunohistochemistry

The deparaffinized sections were rehydrated and heated in sodium citrate buffer for antigen retrieval. The samples were permeabilized, blocked with 0.15% saponin (Sigma), 2% bovine serum albumin (Sigma) and 5% normal goat serum (Gibco), then incubated with primary antibodies (Supplemental Table S6) followed by fluorescein-conjugated IgG secondary antibody (Jackson ImmunoRes, West Grove, PA, USA) and 4,6-diamidino-2-phenylindole nuclear staining and fluorescent signal was visualized under fluorescence microscopy (Zeiss, Oberkochen, Germany).

#### Western blotting and band densitometry

Corneal stromal lysates were denatured in 8 M urea/2% SDS buffer with 1% glycerol, resolved by 4–20% SDS-PAGE (BioRad) and transferred onto nitrocellulose membrane (BioRad). After blocking with 5% nonfat milk (Sigma), the nitrocellulose membrane was incubated with primary antibodies (Supplemental Table S6), followed by appropriate horseradish peroxidase-conjugated Ig secondary antibodies (Jackson ImmunoRes). Staining signals were revealed by enhanced chemiluminescence (ThermoFisher, Waltham, MA, USA) and captured using ChemiDoc XRS gel imaging system (BioRad) as grey-scale high-resolution images. Band densitometry was performed by Quantity One 1D analysis (BioRad) and target protein expression was calculated by band density subtracted with background, then normalized to the intensity of Ponceau S (Sigma) stained total protein profile.

## Supplementary information

Supplemental Information

Supplemental data file S1

Supplemental data file S2

## Data Availability

All data are included in the text, supplemental information and Supplemental Data Files.
