# Supplementary material for: Differential epithelial and stromal protein profiles in cone and non-cone regions of keratoconus corneas
Source: Sci Rep. 2019 Feb 27;9:2965. doi: 10.1038/s41598-019-39182-6 (PMC6393548; doi:10.1038/s41598-019-39182-6)
Supplement: Supplementary file 1 — Supplemental Information [file 41598_2019_39182_MOESM1_ESM.pdf]

**Differential epithelial and stromal protein profiles in cone and non-cone regions of keratoconus corneas**

Gary Hin-Fai Yam, Matthias Fuest, Lei Zhou, Yu-Chi Liu, Lu Deng, Anita Sook-Yee Chan, Hon Shing Ong, Wei-Boon Khor, Marcus Ang, Jodhbir S Mehta

**Supplemental Information**

**Experimental Procedures**

***Quantitative proteomics (SWATH-MS analysis)***

*Sample processing* - Protein samples (at constant 100 μg protein loads, to eliminate quantitative changes due to the corneal volume variation in the thinned cone region compared to the relatively thicker non-cone region) were acetone-precipitated followed by dissolution in lysis buffer (200 mM Tris-HCl pH 8.5, 10 mM EDTA, 4% SDS). Proteins in 5 mM TCEP (Tris 2-carboxyethyl phosphine hydrochloride, Sigma) were denatured at 60°C and passed through 3 kDa cut-off membrane. Retained proteins were washed with 75% urea/Tris-HCl and alkylated in 15 mM iodoacetamide (Sigma). Trypsin-digested peptides were then desalted prior to LC-MS/MS analysis.

*LC-MS/MS* – peptide samples reconstituted in loading buffer (ACN/water with 0.1% formic acid, 2/98 vol/vol) were applied to SGE Reprosil C18-AQ trap column at 10 μl/min and washed for 5 min with loading buffer. The system was switched in line to C18 analytical capillary column connected to a spray tip (New Objectives, Woburn, MA), which was directly coupled with the nano-spray interface into AB Sciex 6600 tripleTOF mass spectrometer. The mobile phases were: solvent A: loading buffer, and solvent B: water/ACN, 2/98 vol/vol, with 0.1% formic acid. A step linear gradient (in total of 2 hr LC run) of mobile phase (solvent B) from 5 to 10% over 0.5 minute, 10 to 20% for 89.5 minutes, 20 to 28% for 30 minutes, 28 to 45% for 10 minutes and lastly, 45 to 80% over 5 min at flow rate of 400 nl/min was utilized.

***Information dependent acquisition (IDA) analysis for building ion library* -** The instrumentation settings were: Ionspray Voltage Floating = 2100 V, curtain gas = 30, ion source gas 1 = 8, interface heater temperature = 125, declustering potential = 80 V and Nebuliser current = 3 for nitrogen gas. All data were acquired using IDA mode (high sensitivity) with Analyst TF1.7 (AB Sciex, Singapore). For IDA parameters, 0.25 sec

TOF MS survey scan in mass range of 350-1800 were followed by product ion scan of 0.05 sec in the mass range of 100-1800. “Rolling collision energy” was used in IDA collision energy setting. Switching criteria were set to ions *m*/*z* >350 and <1800 with charge state of 2-5, maximum number of candidate ions to monitor per cycle was 50 spectra and an abundance threshold of >125 counts. For SWATH acquisition, 100 variable windows between m/z 350-1800 at 30 msec per SWATH window were used.

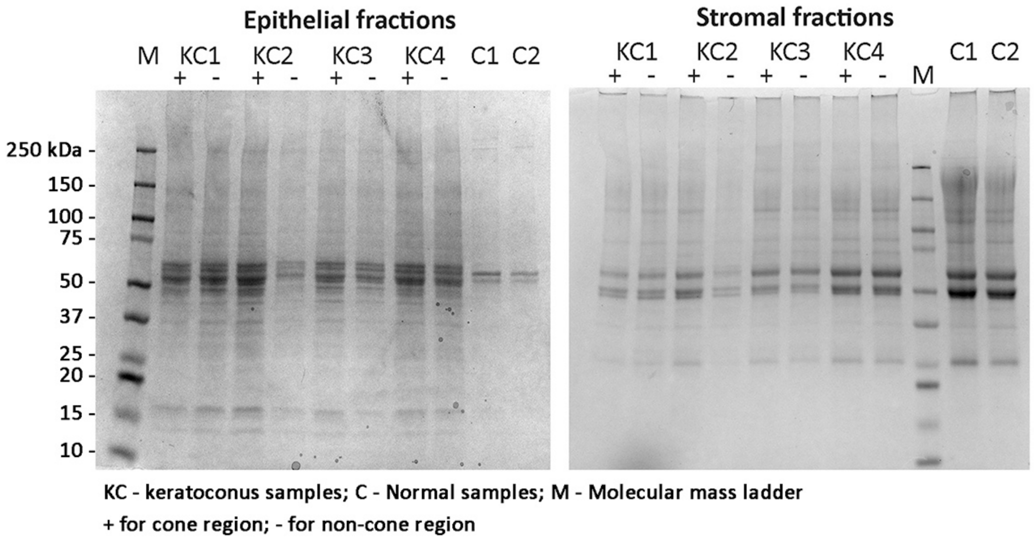


**Supplemental Figure S1.** SDS-soluble proteins extracted from KC and normal corneal samples. Epithelial and stromal fractions from 4 KC samples with separated cone (+) and non-cone (-) regions. The proteins (20 μg) were resolved by 4-20% SDS-PAGE followed by Coomassie Brilliant Blue R250 visualization.

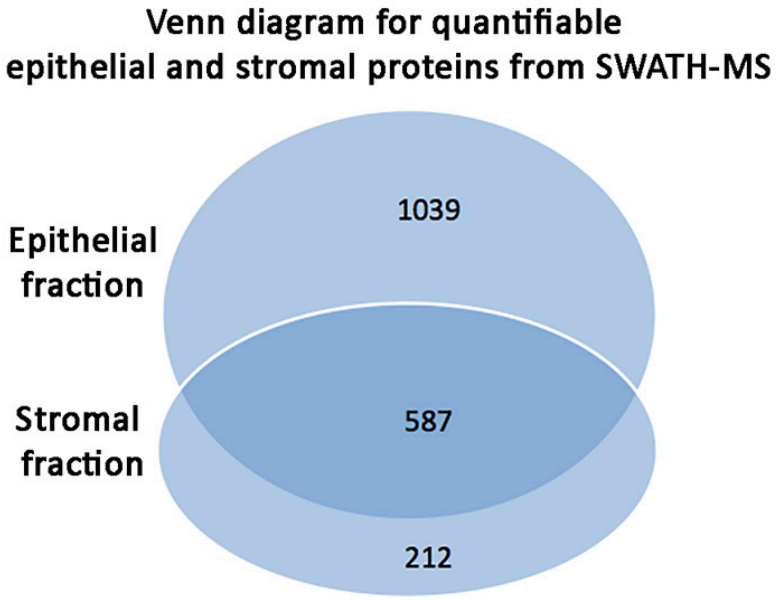


**Supplemental Figure S2.** Venn diagram for quantifiable epithelial and stromal proteins by SWATH-MS.

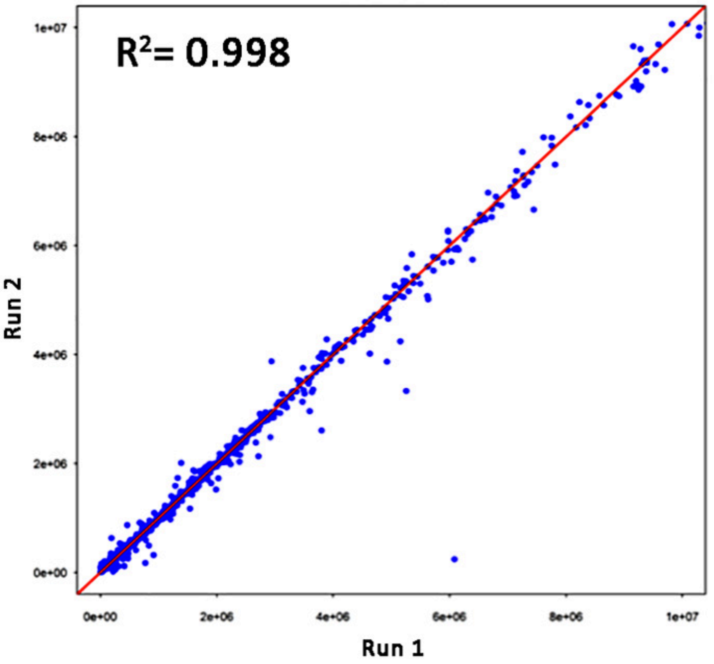


**Supplemental Figure S3.** Reproducibility of quantitation between two technical runs in SWATH-MS using a standard yeast digest sample. Excellent correlation was achieved between two technical runs (correlation efficiency $R^2$ = 0.998).

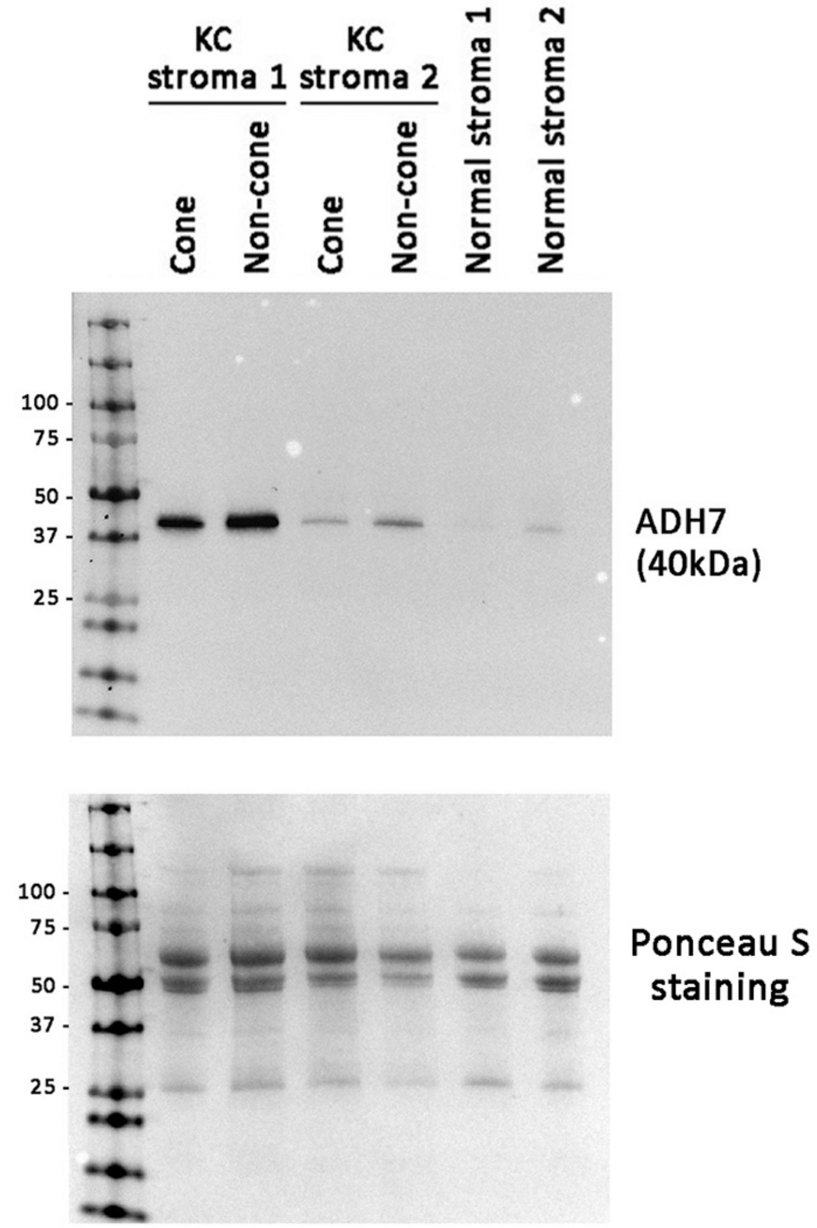


**Supplemental Figure S4.** Full length gel images of western blot data for ADH7 and overall gel pattern revealed by Ponceau S staining as shown in Fig. 6C.

**Supplemental Table S1. Enriched Gene Ontology terms and KEGG pathways identified for differentially expressed proteins between KC cone and normal corneal epithelial samples.** The biological events were ranked using enrichment scores. *Adjusted Benjamini *p*<0.05 represents statistical significance.

| | Enrichment scores | Biological events | Proteins (UniPro Accession no.) | *p* |
|---|---|---|---|---|
| ***(A) Enriched GO terms (DAVID) (non-cone versus normal CE)*** | | | | |
| 1 | 20.1 | GO:0098609~Cell-cell adhesion | Q99439, Q9UGI8, Q13596, Q7L1Q6, Q9UHD8, Q9HC38, Q9BR76, P37802, Q9Y6E2, P31946, P43487, O14618, Q9C0C2, Q9UH65, P30041, P55196, P40121, P07737, Q7KZF4, O95372, P31949, Q15907, P61313, P50402, Q9H3U1, Q99497, Q06830, Q07157, Q9P0L0, P35613, Q9H2G2, Q14677, O75369, Q92522, P28072, O95292, P47756, Q96HC4, O60664, Q02878, Q9NQC3, P53367, Q15365 | 5E-16* |
| 2 | 4.27 | GO:0006120~ Mitochondrial electron transport, NADH to ubiquinone | O43674, O14561, O75489, Q9NX14, O00217, P56556, P09622, O43678, O75380 | 0.013* |
| 3 | 3.87 | GO:0006412~ Translation | P42766, P46779, P39019, P26639, Q969Q0, P62979, P62899, Q9NSD9, P62917, P62266, Q02878, P42677, P18077, P61313, P62851, P46782, P23381 | 0.194 |
| 4 | 3.87 | GO:0000184~ Nuclear-transcribed mRNA catabolic process, nonsense-mediated decay | Q15287, P42766, P46779, P39019, P62979, P62899, P67775, P62917, P62266, Q02878, P42677, P18077, P61313, Q92900, P62851, P46782 | 5.1E-04* |
| 5 | 3.34 | GO:0045454~Cell redox homeostasis | P02788, P07203, P09622, Q13162, P30041, P13667, O95831, P35754, P30044, P10599, Q16881, O76003, Q9BQE4, Q06830, Q13423 | 1.9E-05* |
| 6 | 3.11 | GO:0000398~ mRNA splicing, via spliceosome | Q15287, P52597, P26368, P51991, Q8IYB3, O75533, P62312, Q13435, Q9H2H8, P35637, O43143, P09012, P08621, Q6P2Q9, Q9Y3C6, Q15459, Q15365, Q9Y4Z0 | 0.032* |
| 7 | 2.96 | GO:0006094~ Gluconeogenesis | P40925, P60174, P09467, P04406, P40926, P18669, P00558, P06744, O00757 | 0.006* |
| 8 | 2.81 | GO:0055114~ Oxidation-reduction process | P00441, O14618, Q53TN4, Q13162, P16152, P30838, O95831, P30041, P30044, Q06830, Q13423, O75911, P40394, P00352, P56556, Q6IAN0, Q9Y2Q3, P99999, P35754, P04179, | 0.395 |

| | | | | |
|---|---|---|---|---|
| 9 | 2.587 | GO:0005525~GTP binding | P10599, P52209, Q16881, P07919, Q7Z4W1, P14550, Q9UBQ7, O76003 P68104, P07437, Q9NR31, P61204, Q9UHD8, Q6IQ22, P36405, P61020, P68371, P51148, P11233, P20339, P11234, P21980, O00429, Q96I99, Q9BQE3, Q92930, P57735, Q13885, Q15907, Q9NRW1 | 0.137 |
| 10 | 2.203 | GO:0010951~ Negative regulation of endopeptidase activity | P04080, P30740, P12111, P31025, P30086, P05067, P01011, O43278, Q92530, P36955, P01008, P36952, P01024 | 0.028* |
| ***(B) KEGG pathways (DAVID)*** | | | | |
| 1 | 5.324 | hsa00190: Oxidative phosphorylation | O43674, O14561, P14406, P14854, P08574, O75489, Q9NX14, P56385, O00217, P56556, O43678, Q15181, Q93050, P13073, P10606, P30049, P07919, O75380 | 0.002* |
| 2 | 5.324 | hsa05012: Parkinson's disease | O43674, O14561, P14406, P68036, P14854, P08574, O75489, Q9NX14, O00217, P56556, O43678, P99999, P13073, P10606, P30049, P07919, P42574, O75380, Q99497 | 0.002* |
| 3 | 3.486 | hsa03010: Ribosome | P42766, P46779, P39019, Q969Q0, P62979, P62899, P62917, P62266, Q02878, P42677, P18077, P61313, P62851, P46782 | 0.082 |
| 4 | 3.113 | hsa03040: Spliceosome | P26368, O14776, P51991, O75533, Q13435, P62312, O43143, P09012, P08621, Q6P2Q9, Q9Y3C6, Q15459, Q15365, Q9Y4Z0 | 0.072 |
| 5 | 2.067 | hsa00630: Glycosylate and decarboxylase metabolism | P40925, P40926, Q9BWD1, P34896, Q9UBQ7, P23434, P09622 | 0.015* |

**Supplemental Table S2. Enriched Gene Ontology terms and KEGG pathways identified for differentially expressed proteins between KC non-cone and normal corneal epithelial samples.** The biological events were ranked using enrichment scores. *Adjusted Benjamini $p<0.05$ represents statistical significance.

| | Enrichment scores | Biological events | Proteins (UniPro As session no.) | *p* |
|---|---|---|---|---|
| ***(A) Enriched GO terms (DAVID) (non-cone versus normal CE)*** | | | | |
| 1 | 17.54 | GO:0098609~ Cell-cell adhesion | Q9UGI8, O00151, Q13596, Q9UHD8, Q9HC38, Q9BR76, P37802, O14618, Q9UH65, P30041, P07737, P40121, Q7KZF4, O95372, O95817, P47914, P62826, Q9UNF0, P61313, Q9H3U1, Q99497, Q06830, P53990, Q9P0L0, Q7Z2W4, Q92616, P00338, Q92817, P31939, O00567, O75369, O95292, Q96HC4, O60664, P63244, P14618, Q9NQC3 | 1.2E-14* |
| 2 | 5.32 | GO:0006120~ Mitochondrial electron transport, NADH to ubiquinone | O43674, O14561, O75489, Q9P0J0, Q9NX14, P56556, O43678, O75380, Q16795 | 0.003* |
| 3 | 3.71 | GO:0006094~ Gluconeogenesis | P40925, P60174, P09467, P04406, P40926, P53007, P18669, P00558, P06744 | 0.003* |
| 4 | 3.23 | GO:0006412~ Translation | P60866, P53007, P42766, P46779, Q92616, Q969Q0, P62979, P62899, P62861, E9PAV3, Q9NSD9, P62841, P47914, P42677, P49591, P61313, P46782 | 0.003* |
| 5 | 3.13 | GO:0051287~ NAD binding | O43865, P40925, P04406, P00338, Q9UBQ7, P07195 | 0.083 |
| 6 | 3.04 | GO:0051082~ Unfolded protein binding | P62937, Q16543, Q9UHV9, Q99471, O60925, Q9UBS4, Q9NQP4, P68371 | 0.319 |
| 7 | 2.72 | GO:0000302~Response to ROS | P99999, P30041, P30044, P09211, P04179, P10599, Q16881, O14618, P05090, Q06830 | 1.6E-04* |
| 8 | 2.07 | GO:0015629~Actin cytoskeleton | P20339, P61160, Q9UHD8, O15231, O14776, Q9ULV4, Q96HC4, P07305, Q9BR76, O43707, P23528, O75369, P60981 | 0.046* |
| ***(B) KEGG pathways (DAVID)*** | | | | |
| 1 | 5.32 | hsa00190: Oxidative phosphorylation | O43674, O14561, Q9H2U2, P14854, P08574, O75489, Q9P0J0, Q9NX14, P56385, P56556, O43678, P21283, Q15181, Q93050, P13073, P10606, P30049, P07919, P00403, P36543, O75380, Q16795 | 1.4E-07* |

| | | | | |
|---|---|---|---|---|
| 2 | 5.32 | hsa05012: Parkinson's disease | O43674, O14561, P68036, P14854, P08574, O75489, Q9P0J0, Q9NX14, P56556, O43678, P99999, P13073, P10606, P30049, P07919, P00403, Q16795, O75380, Q99497 | 4.6E-05* |
| 3 | 3.71 | hsa00010: Glycolysis / Gluconeogenesis | P60174, P09467, P04406, P40394, P18669, P00338, P06744, P07195, P30838, P00558, P08237, P14550, P14618 | 6.9E-05* |
| 4 | 3.23 | hsa03010: Ribosome | P60866, P62979, P62861, P62899, P42766, P46779, P62841, P47914, P42677, P61313, Q969Q0, P46782 | 0.069 |
| 5 | 3.13 | hsa00270: Cysteine and methionine metabolism | O43865, P25325, P40925, P40926, P31153, P00338, P07195 | 0.027* |
| 6 | 3.13 | hsa00620: Pyruvate metabolism | P40925, P40926, P00338, Q04760, Q9UBQ7, P14618, P07195 | 0.033* |

**Supplemental Table S3**. **Enriched Gene Ontology terms and KEGG pathways identified for differentially expressed proteins between KC cone and normal corneal stromal samples**. The biological events were ranked using enrichment scores. All had adjusted Benjamini *p*<0.05 representing statistical significance.

| | Enrichment scores | Biological events | Proteins (UniPro As session no.) | *p* |
|---|---|---|---|---|
| ***(A) Enriched GO terms (DAVID) (non-cone versus normal CE)*** | | | | |
| 1 | 10.72 | GO:0098609~Cell-cell adhesion | Q7L1Q6, P31947, P25685, P37802, Q00341, P30041, P52907, P40121, P15311, O95372, P50990, Q9Y265, P63104, P42167, Q9Y490, Q02487, P21333, Q14134, P14923, Q9NTK5, Q15417, Q05682, Q6IBS0, Q15691, P23229, Q14247 | 3.97E-11 |
| 2 | 7.88 | GO:0005576~ Extracellular region | P01031, Q14574, Q06828, P01714, P14780, P16035, P68366, P18428, P06309, P08697, P01593, P02787, P40394, Q9Y490, P02748, P02747, P21333, Q9Y240, P02743, Q6UWY5, P08123, P02649, P01742, P29622, P00746, P35443, P01781, P00441, P27797, P09871, P41222, P36980, P84243, P52907, O43866, P20774, Q14116, P01776, P01773, Q92743, P10745, P02652, P02760, P02671, P01861, P02768, P02766, P02452, Q9UBX5, P31025, P23083, P51888, P05154, Q12805, P21810, Q08380, P09429, P06316, P0C0L4, P01763 | 1.8E-15 |
| 3 | 5.37 | GO:0006457~ Protein folding | P40227, P78371, Q8NBS9, P27797, O95302, P25685, Q9Y230, P02743, P49368, P10599, P48643, P50990, P50991, P26885 | 3.2E-11 |
| 4 | 5.11 | GO:0006958~ Complement activation, classical pathway | P01593, P01781, P01031, P01861, P01871, P02748, P02747, P01714, Q07021, P09871, P06309, P01776, P01773, P06316, P01880, P01742, P0C0L4, P01763 | 5.1E-04 |
| 5 | 5.11 | GO:0038095~Fc ε-receptor signaling pathway | P01781, P01593, P25789, P62837, Q06323, P01714, P63000, P62158, P06309, P01776, P01773, P01742, P06316, P01763 | 1.8E-04 |
| 6 | 3.02 | GO:0005525~GTP binding | P68104, P36405, P30520, Q9NVA2, Q9NTK5, P61019, P68371, P63000, P18085, P21980, P68366, P61586, Q9BQE3, Q13885, Q9BUF5 | 0.035 |
| 7 | 2.84 | GO:0005882~ Intermediate filament | P02545, P22392, P15924, P13645, P12035, P02533, P35908, P14923 | 0.011 |
| 8 | 2.69 | GO:0010951~ | P01031, P16035, P05154, O95428, | 6.8E-04 |

| | | | | |
|---|---|---|---|---|
| | | Negative regulation of endopeptidase activity | P36952, P08697, P31025, P35237, P0C0L4, P29622, P02760 | |
| 9 | 2.43 | GO:0030574~ Collagen catabolic process | P12110, P12111, P12109, P02452, P14780, P08123 | 0.003 |
| 10 | 2.37 | GO:0000302~ Response to reactive oxygen species | P30041, P02649, P30044, P09211, P00441, P10599 | 0.012 |
| ***(B) KEGG pathways (DAVID)*** | | | | |
| 1 | 2.9 | hsa04610: Complement and coagulation cascades | P01031, P02671, P05154, P02748, P02747, P08697, P09871, P0C0L4, P00746 | 0.006 |
| 2 | 2.02 | hsa00010: Glycolysis / Gluconeogenesis | P09972, P09467, P40394, P18669, P49189, P00325, P17858 | 0.046 |

**Supplemental Table S4**. **Enriched Gene Ontology terms and KEGG pathways identified for differentially expressed proteins between KC non-cone and normal corneal stromal samples**. The biological events were ranked using enrichment scores. All had adjusted Benjamini *p*<0.05 representing statistical significance.

| | Enrichment scores | Biological events | Proteins (UniPro As session no.) | *p* |
|---|---|---|---|---|
| ***(A) Enriched GO terms (DAVID) (non-cone versus normal CE)*** | | | | |
| 1 | 9.32 | GO:0098609~Cell-cell adhesion | Q7L1Q6, P61026, P31947, Q14134, P37802, P31946, P43487, P40121, P52907, O60763, Q15417, Q05682, O95372, P50990, P14618, P09758, Q9Y265, P63104 | 2.9E-05 |
| 2 | 6.51 | GO:0006958~ Complement activation, classical pathway | P01593, P01861, P01871, P02748, P01714, Q07021, P01611, P01601, P09871, P04433, P04220, P01857, P01623, P06309, P01764, P01773, P01880, P01742, P0C0L4, P06681 | 7.6E-13 |
| 3 | 6.51 | GO:0038096~Fc-γ receptor signaling pathway involved in phagocytosis | P01593, P01861, O15145, P01714, P01611, P01601, O15511, P04433, P01857, P01623, P06309, P01764, P01773, P01742 | 5.4E-06 |
| 4 | 6.51 | GO:0006955~ Immune response | P01593, P02748, P01714, Q07021, P01611, P01601, P23083, P04433, P22413, P01623, P06309, P01764, P18510, Q14116, P01773, P01880, P01742 | 0.01 |
| 5 | 5.55 | GO:0005576~ Extracellular region | Q06828, P01714, P01611, P14780, Q9ULZ3, P16035, P12830, P01857, P18428, P68366, P06309, P08697, P55058, P06681, P01593, Q13751, P40394, P02788, P12111, P02748, P01601, P21333, Q9Y240, P02743, Q6UWY5, P08123, P04433, P02649, P24592, Q99969, P01742, P29622, P00746, P00441, P27797, P09871, P68871, P36980, P52907, O43866, Q14116, P01773, Q92743, P02760, P02671, P01861, P12110, P02766, P02452, P31025, P23083, P51888, P05154, P12109, P01623, P01764, Q12805, P21810, P09429, P0C0L4 | 1.8E-12 |
| 6 | 3.44 | GO:0016491~ Oxidoreductase activity | Q06828, P01714, P01611, P14780, Q9ULZ3, P16035, P12830, P01857, P18428, P68366, P06309, P08697, P55058, P06681, P01593, Q13751, P40394, P02788, P12111, P02748, P01601, P21333, Q9Y240, P02743, Q6UWY5, P08123, P04433, P02649, P24592, Q99969, P01742, P29622, | 1.8E-12 |

| | | | | |
|---|---|---|---|---|
| | | | P00746, P00441, P27797, P09871, P68871, P36980, P52907, O43866, Q14116, P01773, Q92743, P02760, P02671, P01861, P12110, P02766, P02452, P31025, P23083, P51888, P05154, P12109, P01623, P01764, Q12805, P21810, P09429, P0C0L4 | |
| 7 | 3.11 | GO:0034987~ Immunoglobulin receptor binding | P01861, P04220, P01871, P01857, P01764, P01601, P01880 | 0.001 |
| ***(B) KEGG pathways (DAVID)*** | | | | |
| 1 | 2.46 | hsa00010: Glycolysis / Gluconeogenesis | P09972, P09467, P40394, P18669, P00325, P09104, P14618, P17858 | 0.007 |
| 2 | 2.43 | hsa04512: ECM-receptor interaction | P12110, Q13751, P12111, P05556, P12109, P02452, P23229, P08123 | 0.023 |

**Supplemental Table S5. Patient information for archived KC and non-KC specimens**

| | | | | |
|---|---|---|---|---|
| ***(A) Archived KC specimens*** | | | | |
| **Patient** | **Age** | **Sex** | **Diagnosis** | **Surgery** |
| 1 | 21 | M | Advanced KC | DALK |
| 2 | 27 | M | Advanced KC | DALK |
| 3 | 21 | M | Advanced KC | DALK |
| 4 | 54 | F | Advanced KC | DALK |
| ***(B) Archived non-KC specimens*** | | | | |
| 1 | 87 | F | Corneal scar | PK |
| 2 | 42 | F | Phytencular inflammatory corneal scar | DALK |
| 3 | 60 | M | HSV keratitis–related corneal scaring | DALK |
| 4 | 44 | M | Previous fungal keratitis with scaring and inflammation | DALK |
| ***(C) Normal corneal specimens*** | | | | |
| **Patient** | **Age** | **Sex** | **Cause of Death** | |
| 1 | 37 | F | Acute cardiac crisis | |
| 2 | 59 | M | Coronary artery disease | |

M – male; F – female; DALK - deep anterior lamellar keratoplasty; PK – penetrating keratoplasty

**Supplemental Table S6. Primary antibody information**

| **Antibody (clone)** | **Company (Catalogue No.)** | **Applications** | **Working concentration** |
|---|---|---|---|
| NDUFB11 (G-4) | Santa Cruz (sc-374369) | Immunohistochemistry | 1.5 μg/ml |
| ADH7 | Proteintech (23425) | Immunohistochemistry<br>Western blotting | 0.5 μg/ml |
| STEAP4 | ThermoFisher (PA5-62054) | Immunohistochemistry | 2 μg/ml |
| IL1RN | Sigma-Aldrich (HPA001482) | Immunohistochemistry | 2 μg/ml |
| β-Actin-horseradish peroxidase conjugate | Sigma (A3854) | Western blotting | 0.5 μg/ml |

**Supplemental Table S7.** Reported functions of dysregulated proteins between KC cone and non-cone epithelial and stromal samples and potential link to KC pathogenesis

| | Protein, UniPro No. | Cone vs Non-cone (fold change) | Reported functions and potential link to keratoconus pathogenesis | Ref |
|---|---|---|---|---|
| ***(A) Deregulated epithelial proteins in cone versus non-cone regions*** | | | | |
| 1 | NADH: Ubiquinone oxidoreductase subunit B11, NDUFB11, Q9NX14 | Up (24±17) | An integral inner mitochondrial membrane protein transfers electron from NADH to ubiquinone. Reported mutations link to microophthalmia with epithelial defects | (1, 2) |
| 2 | Glucosidase a, acid, GAA, P10253 | Up (6.7±3) | Glycogen degradation | (3) |
| 3 | Nicotinamide nucleotide transhydrogenase, NTT, Q13423 | Up (2.5±1) | An integral inner mitochondrial membrane protein transfers hydrides between NAD(H) and NADP(+) to proton translocation | (1) |
| 4 | DNA fragmentation factor subunit a, DFFA, O00273 | Up (3±1.5) | A substrate of caspase-3 to trigger DNA fragmentation | (4) |
| 5 | Ribosomal protein L28, RPL28, P46779 | Up (3±1.5) | A component of 60S subunit to regulate translation | (5) |
| 6 | Insulin-like growth factor binding protein 6, IGFBP6, P24592 | Down (0.3±0.03) | IGF metabolism to regulates cell growth, survival and apoptosis | (6) |
| 7 | Fumarate hydratase, FH, P07954 | Down (0.2±0.1) | Involves in enzymatic reaction in tricarboxylic acid cycle | (7) |
| 8 | Glutaminase, GLS, O94925 | Down (0.3±0.1) | Regulates energy production and antioxidant defense | (8) |
| 9 | Translocase of outer mitochondrial membrane 22, | Down (0.5±0.2) | Imports cytosolically synthesized mitochondrial preproteins for mitochondria homeostasis | (9) |

| | | | | |
|---|---|---|---|---|
| | TOMM22, Q9NS69 | | | |
| 10 | Eukaryotic translation initiation factor 3 subunit G, EIF3G, O75821 | Down (0.5±0.1) | Translation initiation | (10) |
| 11 | Eukaryotic translation initiation factor 2β, EIF2S2, P20042 | Down (0.5±0.2) | Forms ternary complex with GTP and initiator tRNA in early steps of protein synthesis | |
| 12 | ADP ribosylation factor interacting protein 1, ARFIP1, P53367 | Down (0.4±0.1) | Regulates post-translational modifications | (11) |
| 13 | Syntaxin 4, STX4, Q12846 | Down (0.4±0.2) | Vesicle transport to plasma membrane and cytokine signaling in immune system | (12) |
| 14 | Platelet activating factor acetylhydrolase 1b regulatory subunit 1, PAFAH1B1, P40394 | Down (0.5±0.1) | Involves in microtubule polymerization for maintaining intracellular organelle integrity, intracellular trafficking, nucleokinesis and cell locomotion | (13) |
| 15 | Niemann-Pick type C2, NPC2, P61916 | Down (0.4±0.2) | Regulates cholesterol transport through the late endosomal/lysosomal system and proper clearance of autophagosomes. Dysregulated autophagy due to oxidative stress has been reported in KC epithelia. | (14, 15) |
| 16 | Prefoldin subunit 4, PFDN4, Q9NQP4 | Down (0.4±0.1) | A component of molecular chaperone complex involved in protein folding and prevention of protein aggregation, which is associated to ER stress and oxidative stress responses | (16, 17) |
| 17 | ADAM metallopeptida | Down (0.5±0.1) | A cell surface protein with protease activity in cleaving various extracellular proteins | (18) |

| | | | | |
|---|---|---|---|---|
| | se domain 10, ADAM10, O14672 | | like TNFa and E-cadherin and has been implicated in EGFR and IL6 signaling, the latter is an inflammatory cytokine detected in KC epithelia. | |
| *(B) Deregulated stromal proteins in cone versus non-cone regions* | | | | |
| 1 | Threonyl-tRNA synthetase, SYTC, P26639 | Up (5±3) | tRNA aminoacylation for protein translation; immune response and angiogenesis | |
| 2 | RuvB like AAA-ATPase2, RUVBL2, Q9Y230 | Up (2.4±1) | Acts as DNA helicase for homologous recombination and DNA break repairs; involves in ER-associated degradation pathway in regulating ER stress response; links to protein folding in stromal repair and ROS-related activities in KC | (19) |
| 3 | Secretion-associated Ras-related GTPase 1A, SAR1A, Q9NR31 | Down (0.4±0.2) | Involves in endoplasmic reticulum (ER)-Golgi transport for protein post-translational processing and modifications | (20) |
| 4 | Tumor associated calcium signal transducer 2, TACSTD2, P09758 | Down (0.3±0.1) | Reported for familial gelatinous drop-like corneal dystrophy with amyloid deposits. Its association to secondary corneal amyloidosis and inflammatory pathways that occur in keratoconus needs to be verified. | (21-23) |
| 5 | Vitrin, VIT, Q6UXI7 | Down (0.4±0.2) | Involves in glycosaminoglycan binding during cell adhesion and migration; morpholino knockdown increased apoptosis | (24) |
| 6 | STEAP4 metalloreductase, Q687X5 | Down (0.5±0.3) | A mitochondrial iron reductase participating in systemic metabolic homeostasis, integrating inflammatory and metabolic responses; relates to mitochondrial damages and oxidative stress | (25) |
| 7 | Phosphoglycerate dehydrogenase, PHDGH, O43175 | Down (0.5±0.3) | Involves in NAD reduction for electron transfer and oxidoreductase activities. Reported variants in association with corneal epithelial and stromal thickness | (26, 27) |
| 8 | ATP binding cassette subfamily B11, ABCB11, O95342 | Down (0.4±0.1) | Contains transporter and ATPase activities involved in multidrug resistance and bile salt secretion | |
| 9 | Splicing factor 3b subunit 3, SF3B3, Q15393 | Down (0.5±0.3) | mRNA splicing | (28) |
| 10 | Basic leucine zipper and W2 domain 1, | Down (0.5±0.2) | Regulates histone H4 gene transcription | (29) |

| | | | | |
|---|---|---|---|---|
| | BZW1, Q7L1Q6 | | | |
| 11 | H2A histone family member J, H2AJ, Q9BTM1 | Down (0.4±0.2) | Epigenetic regulation | (29) |
| 12 | Macrophage migrating inhibitory factor, MIF, P14174 | Down (0.5±0.2) | An inflammation-regulatory cytokine released upon corneal injury | (30) |
| 13 | (NAD(P)H quinone dehydrogenase 1, NQO1, P15559 | Down (0.4±0.2) | A downstream cytoprotective effector of NOX4 and ROS signaling against oxidative stress in corneal diseases | (31) |
| 14 | Alcohol dehydrogenase 7, ADH7, P40394 | Down (0.5±0.1) | Possesses 11-cis-retinol dehydrogenase activity in photo-pigment regeneration; a defense role against oxidative damage from alcohols and aldehydes (by reducing 4-hydrozynonenal, a lipid peroxidation cytotoxic product) | (32) |

**References for Supplemental Information**


1. Meimaridou, E., Kowalczyk, J., Guasti, L., Hughes, C. R., Wagner, F., Frommolt, P., Nurnberg, P., Mann, N. P., Banerjee, R., Saka, H. N., Chapple, J. P., King, P. J., Clark, A. J., and Metherell, L. A. (2012) Mutations in NNT encoding nicotinamide nucleotide transhydrogenase cause familial glucocorticoid deficiency. *Nature Genet* **44**, 740-742

2. van Rahden, V. A., Fernandez-Vizarra, E., Alawi, M., Brand, K., Fellmann, F., Horn, D., Zeviani, M., and Kutsche, K. (2015) Mutations in NDUFB11, encoding a complex I component of the mitochondrial respiratory chain, cause microphthalmia with linear skin defects syndrome. *Am J Human Genet* **96**, 640-650

3. Wan, L., Lee, C. C., Hsu, C. M., Hwu, W. L., Yang, C. C., Tsai, C. H., and Tsai, F. J. (2008) Identification of eight novel mutations of the acid alpha-glucosidase gene causing the infantile or juvenile form of glycogen storage disease type II. *J Neurol* **255**, 831-838

4. Liu, X., Zou, H., Slaughter, C., and Wang, X. (1997) DFF, a heterodimeric protein that functions downstream of caspase-3 to trigger DNA fragmentation during apoptosis. *Cell* **89**, 175-184

5. Frigerio, J. M., Dagorn, J. C., and Iovanna, J. L. (1995) Cloning, sequencing and expression of the L5, L21, L27a, L28, S5, S9, S10 and S29 human ribosomal protein mRNAs. *Biochim Biophys Acta* **1262**, 64-68

6. Neumann, G. M., Marinaro, J. A., and Bach, L. A. (1998) Identification of O-glycosylation sites and partial characterization of carbohydrate structure and disulfide linkages of human insulin-like growth factor binding protein 6. *Biochemistry* **37**, 6572-6585

7. Toro, J. R., Nickerson, M. L., Wei, M. H., Warren, M. B., Glenn, G. M., Turner, M. L., Stewart, L., Duray, P., Tourre, O., Sharma, N., Choyke, P., Stratton, P., Merino, M., Walther, M. M., Linehan, W. M., Schmidt, L. S., and Zbar, B. (2003) Mutations in the fumarate hydratase gene cause hereditary leiomyomatosis and renal cell cancer in families in North America. *Am J Human Genet* **73**, 95-106

8. Hu, W., Zhang, C., Wu, R., Sun, Y., Levine, A., and Feng, Z. (2010) Glutaminase 2, a novel p53 target gene regulating energy metabolism and antioxidant function. *Proc Nat Acad Sci USA* **107**, 7455-7460

9. Yano, M., Hoogenraad, N., Terada, K., and Mori, M. (2000) Identification and functional analysis of human Tom22 for protein import into mitochondria. *Mol Cell Biol* **20**, 7205-7213

10. Lee, A. S., Kranzusch, P. J., Doudna, J. A., and Cate, J. H. (2016) eIF3d is an mRNA cap-binding protein that is required for specialized translation initiation. *Nature* **536**, 96-99

11. Liu, C., and Yu, X. (2015) ADP-ribosyltransferases and poly ADP-ribosylation. *Current protein & peptide science* **16**, 491-501

12. Low, S. H., Vasanji, A., Nanduri, J., He, M., Sharma, N., Koo, M., Drazba, J., and Weimbs, T. (2006) Syntaxins 3 and 4 are concentrated in separate clusters on the plasma membrane before the establishment of cell polarity. *Mol Biol Cell* **17**, 977-989

13. Tanaka, M., Kim, Y. M., Lee, G., Junn, E., Iwatsubo, T., and Mouradian, M. M. (2004) Aggresomes formed by alpha-synuclein and synphilin-1 are cytoprotective. *J Biol Chem* **279**, 4625-4631

14. Elrick, M. J., Yu, T., Chung, C., and Lieberman, A. P. (2012) Impaired proteolysis underlies autophagic dysfunction in Niemann-Pick type C disease. *Human Mol Genet* **21**, 4876-4887

15. Shetty, R., Sharma, A., Pahuja, N., Chevour, P., Padmajan, N., Dhamodaran, K., Jayadev, Ghosh, A., and Nallathambi, J. (2017) Oxidative stress induces dysregulated autophagy in corneal epithelium of keratoconus patients. *PloS One* **12**, e0184628

16. Vainberg, I. E., Lewis, S. A., Rommelaere, H., Ampe, C., Vandekerckhove, J., Klein, H. L., and Cowan, N. J. (1998) Prefoldin, a chaperone that delivers unfolded proteins to cytosolic chaperonin. *Cell* **93**, 863-873

17. Abe, A., Takahashi-Niki, K., Takekoshi, Y., Shimizu, T., Kitaura, H., Maita, H., Iguchi-Ariga, S. M., and Ariga, H. (2013) Prefoldin plays a role as a clearance factor in preventing proteasome inhibitor-induced protein aggregation. *J Biol Chem* **288**, 27764-27776

18. Shetty, R., Ghosh, A., Lim, R. R., Subramani, M., Mihir, K., Reshma, A. R., Ranganath, A., Nagaraj, S., Nuijts, R. M., Beuerman, R., Shetty, R., Das, D., Chaurasia, S. S., Sinha-Roy, A., and Ghosh, A. (2015) Elevated expression of matrix metalloproteinase-9 and inflammatory cytokines in keratoconus patients is inhibited by cyclosporine A. *Invest Ophthalmol Vis Sci* **56**, 738-750

19. Cheng, H. M., Sun, H. Y., Lin, D. P., Chang, H. H., Chen, S. T., Yeh, S. M., Peng, M. L., Tseng, J. K., Su, K. C., Tseng, K. W., Chen, B. Y., Hsiao, C. J., Huang, S. Y., and Cheng, C. Y. (2012) Characterising visual deficits in children of an urban elementary school in Taiwan. *Clin Exp Optom* **95**, 531-537

20. Petrosyan, A., Cheng, P. W., Clemens, D. L., and Casey, C. A. (2015) Downregulation of the small GTPase SAR1A: a key event underlying alcohol-induced Golgi fragmentation in hepatocytes. *Sci Rep* **5**, 17127

21. Jongkhajornpong, P., Lekhanont, K., Ueta, M., Kitazawa, K., Kawasaki, S., and Kinoshita, S. (2015) Novel TACSTD2 mutation in gelatinous drop-like corneal dystrophy. *Human Genome Var* **2**, 15047

22. Cabral-Macias, J., Zenteno, J. C., Ramirez-Miranda, A., Navas, A., Bermudez-Magner, J. A., Boullosa-Grana, V. M., Graue-Hernandez, E. O., and Buentello-Volante, B. (2016) Familial gelatinous drop-Like corneal dystrophy caused by a novel nonsense TACSTD2 mutation. *Cornea* **35**, 987-990

23. Alehabib, E., Jamshidi, J., Ghaedi, H., Emamalizadeh, B., Andarva, M., Daftarian, N., Rezaei Kanavi, M., Mohammadi Torbati, P., Espandar, G., Alinaghi, S., Johari, A. H., Saghally, M., Mohajerani, F., and Darvish, H. (2017) Novel mutations in TACSTD2 gene in families with gelatinous drop-like corneal dystrophy (GDLD). *Int J Mol Cell Med* **6**, 204-211

24. Wang, M. S., Zhang, R. W., Su, L. Y., Li, Y., Peng, M. S., Liu, H. Q., Zeng, L., Irwin, D. M., Du, J. L., Yao, Y. G., Wu, D. D., and Zhang, Y. P. (2016) Positive selection rather than relaxation of functional constraint drives the evolution of vision during chicken domestication. *Cell Res* **26**, 556-573

25. Gordon-Shaag, A., Millodot, M., Shneor, E., and Liu, Y. (2015) The genetic and environmental factors for keratoconus. *BioMed Res Int* **2015**, 795738

26. Mullarky, E., Lucki, N. C., Beheshti Zavareh, R., Anglin, J. L., Gomes, A. P., Nicolay, B. N., Wong, J. C., Christen, S., Takahashi, H., Singh, P. K., Blenis, J., Warren, J. D., Fendt, S. M., Asara, J. M., DeNicola, G. M., Lyssiotis, C. A., Lairson, L. L., and Cantley, L. C. (2016) Identification of a small molecule

inhibitor of 3-phosphoglycerate dehydrogenase to target serine biosynthesis in cancers. *Proc Nat Acad Sci USA* **113**, 1778-1783

27. Koehn, D. R., Meyer, K. J., and Anderson, M. G. (2015) Genetic Evidence for Differential Regulation of Corneal Epithelial and Stromal Thickness. *Invest Ophthalmol Vis Sci* **56**, 5599-5607

28. Chen, K., Xiao, H., Zeng, J., Yu, G., Zhou, H., Huang, C., Yao, W., Xiao, W., Hu, J., Guan, W., Wu, L., Huang, J., Huang, Q., Xu, H., and Ye, Z. (2017) Alternative Splicing of EZH2 pre-mRNA by SF3B3 Contributes to the Tumorigenic Potential of Renal Cancer. *Clin Cancer Res* **23**, 3428-3441

29. Nielsen, K., Hjortdal, J., Pihlmann, M., and Corydon, T. J. (2013) Update on the keratoconus genetics. *Acta Ophthalmol* **91**, 106-113

30. Matsuda, A., Tagawa, Y., Matsuda, H., and Nishihira, J. (1997) Expression of macrophage migration inhibitory factor in corneal wound healing in rats. *Invest Ophthalmol Vis Sci* **38**, 1555-1562

31. Gorrini, C., Harris, I. S., and Mak, T. W. (2013) Modulation of oxidative stress as an anticancer strategy. *Nature Rev Drug Discovery* **12**, 931-947

32. Alvarez, R., Vaz, B., Gronemeyer, H., and de Lera, A. R. (2014) Functions, therapeutic applications, and synthesis of retinoids and carotenoids. *Chem Rev* **114**, 1-125

**Supplemental Data File S1:**

Lists of quantifiable epithelial and stromal proteins from KC samples by SWATH-MS.

**Supplemental Data File S2:**

Lists of up- and down-regulated epithelial and stromal proteins compared among KC cone, non-cone and normal corneal samples. Mean fold changes, SD and P-values are listed for each candidate proteins.
